# Machine learning for refining interpretation of magnetic resonance imaging scans in the management of multiple sclerosis: a narrative review

**DOI:** 10.1098/rsos.241052

**Published:** 2025-01-22

**Authors:** Adam C. Szekely-Kohn, Marco Castellani, Daniel M. Espino, Luca Baronti, Zubair Ahmed, William G. K. Manifold, Michael Douglas

**Affiliations:** ^1^ School of Engineering, University of Birmingham, Edgbaston, Birmingham B15 2TT, UK; ^2^ School of Computer Science, University of Birmingham, Edgbaston, Birmingham B15 2TT, UK; ^3^ University Hospitals Birmingham NHS Foundation Trust, Edgbaston, Birmingham B15 2GW, UK; ^4^ Institute of Inflammation and Ageing, University of Birmingham, Edgbaston, Birmingham B15 2TT, UK; ^5^ The Royal London Hospital, Barts Health NHS Trust, Whitechapel Road, London E1 1FR, UK; ^6^ Department of Neurology, Dudley Group NHS Foundation Trust, Russells Hall Hospital, Birmingham DY1 2HQ, UK; ^7^ School of Life and Health Sciences, Aston University, Birmingham, UK

**Keywords:** artificial intelligence, computational methods, machine learning, magnetic resonance imaging (MRI), multiple sclerosis (MS)

## Abstract

Multiple sclerosis (MS) is an autoimmune disease of the brain and spinal cord with both inflammatory and neurodegenerative features. Although advances in imaging techniques, particularly magnetic resonance imaging (MRI), have improved the process of diagnosis, its cause is unknown, a cure remains elusive and the evidence base to guide treatment is lacking. Computational techniques like machine learning (ML) have started to be used to understand MS. Published MS MRI-based computational studies can be divided into five categories: automated diagnosis; differentiation between lesion types and/or MS stages; differential diagnosis; monitoring and predicting disease progression; and synthetic MRI dataset generation. Collectively, these approaches show promise in assisting with MS diagnosis, monitoring of disease activity and prediction of future progression, all potentially contributing to disease management. Analysis quality using ML is highly dependent on the dataset size and variability used for training. Wider public access would mean larger datasets for experimentation, resulting in higher-quality analysis, permitting for more conclusive research. This narrative review provides an outline of the fundamentals of MS pathology and pathogenesis, diagnostic techniques and data types in computational analysis, as well as collating literature pertaining to the application of computational techniques to MRI towards developing a better understanding of MS.

## 1. Introduction

**Figure 1.**
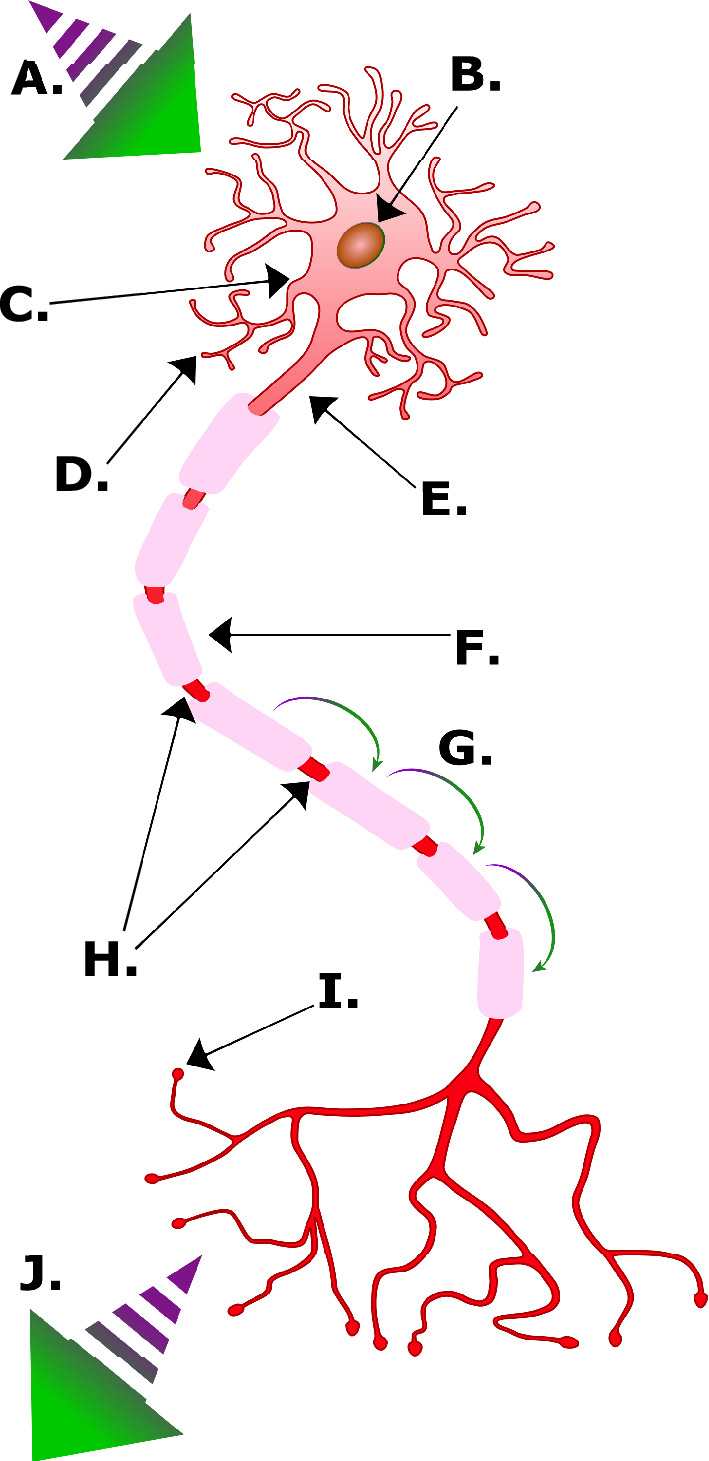
A labelled illustration of a myelinated neural cell. A, nerve signal input; B, cell nucleus; C, soma; D*,* dendrites; E, axon; F, myelin sheath; G, nerve signal; H, nodes of Ranvier; I, synaptic terminals; J, nerve signal output (original image).

A chronic inflammatory condition, multiple sclerosis (MS), is characterized by damage to myelin within the central nervous system (CNS)—demyelination—and the resultant loss of function (see figure 1). Several potential factors have been identified as significant risk factors for MS, including previous infection with Epstein–Barr virus [1,2], geographical latitude [3], month of birth [4] and certain genetic polymorphisms [5,6]. In common with many autoimmune diseases, MS disproportionately affects women, with a female-to-male ratio of 3:1 [7].

Myelin is composed of proteins and lipids and acts to increase the efficiency of signal propagation from a cell body to its dendrites/terminals [8]. Myelin production and maintenance are dependent on oligodendrocytes, a specialized type of glial cell [9]. Several types of inflammatory cells are likely to be involved in the pathogenesis of MS, including CD4^+^ T-lymphocytes, which recognize antigen peptide fragments bound to major histocompatibility complex class II proteins. These antigenic fragments may be derived from a range of autoantigens such as myelin basic proteins, myelin proteolipid protein, myelin oligodendrocyte glycoprotein and myelin-associated glycoprotein [10,11]. T-lymphocyte activation in the peripheral blood or secondary lymphoid tissues results in subsequent crossing of the blood–brain barrier (BBB) and damage within the CNS.

### 1.1. Multiple sclerosis pathology

**Figure 2.**
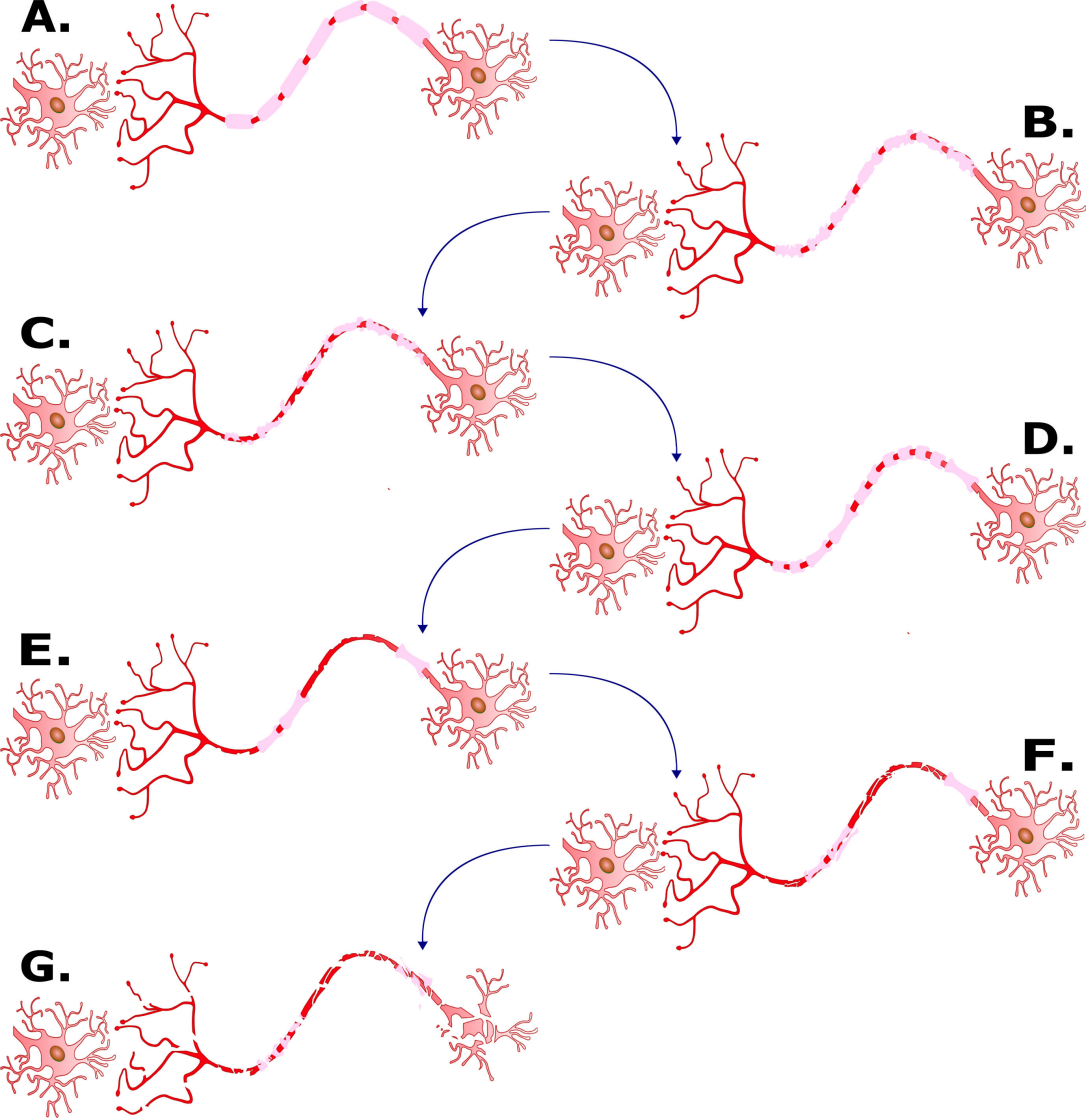
A graphical depiction of the demyelination process. A, healthy neural cell; B, demyelination begins to take place due to malfunctioning immune system; C, myelin is stripped from the axon; D, oligodendrocytes and Schwann cells induce partial remyelination; E, subsequent inflammation event(s) destroys the capacity for remyelination; F, axon begins to degenerate; G, neural cell degenerates and disintegrates (original image).

At a cellular level, MS is fundamentally a demyelinating disease, stripping axons of their myelin sheath, leading to energy deficiencies and ion imbalances in brain tissue [12], ultimately resulting in axonal destruction [13]. This process is depicted in figure 2.

Myelin-specific T-lymphocytes, in conjunction with tissue-resident cells, generate proinflammatory cytokines (such as interleukin-1, interleukin-6, tumour necrosis factor-α and interferon-γ (IFN-γ)) that have local effects including amplification of the inflammatory cascade and increasing influx of other cell populations from the peripheral blood (e.g. B-lymphocytes) [14]. IFN-γ can also cause damage to the oligodendrocytes, limiting remyelination capability [15]. Alongside T-lymphocytes, B-cells produce antibodies that target cell surface proteins expressed on the myelin sheath and oligodendrocytes [16,17]. Amplification of the inflammatory phase is followed by a period of inflammatory resolution, likely to involve additional cell populations such as regulatory T-lymphocytes [18]. As the inflammatory event resolves, tissue restorative and remodelling processes begin (remyelination) but with super-added areas of tissue scarring (gliosis). Over time, remyelination efficiency decreases and gliosis becomes more prominent [19]. The inflammatory processes lead to foci of inflammatory activity within the CNS [20], which can be visualized on magnetic resonance imaging (MRI) and produce clinical correlates depending on their location. The associated symptomatology can include visual disturbance [21] (from optic neuritis or brainstem lesions), limb weakness [22,23] and sensory disturbance [24,25] (from spinal cord lesions).

### 1.2. A brief history of multiple sclerosis

The pathology of MS was first formally documented at the end of the nineteenth century with the first key observations on MS through the work of Carl Frommann [26] and Jean-Martin Charcot [27]. Frommann focused on spinal cord lesions and the axonal destruction present within those tissues [26], whereas the work of Charcot centred on brain lesions, in which he correctly postulated that MS was primarily a demyelinating disease resulting in axonal injury [27]. This view was subsequently supported by the work of Joseph Babinski [28]. The subsequent research of Max Fraenkel and Alfons Jakob highlighted the roles of macrophages during demyelination and axonal destruction [29]. Using an animal model of demyelination, Thomas Rivers was the first to propose that MS was primarily an autoimmune disease and not an infective (particularly viral) condition [30], although evidence for a role for a viral trigger, particularly Epstein–Barr virus, continues to accumulate [1,2,31]. Alongside a primary role for tissue inflammation-mediated demyelination, additional evidence highlights that grey matter damage is also prominent, providing another neurodegenerative element to MS pathology [32].

Imaging techniques, in particular MRI, helped further the understanding of MS. MRI became increasingly important in the diagnosis and monitoring of MS patients from the 1990s onwards, with a steady increase in the range of modalities of MRI used in visualization of various aspects of the disease [33,34]. Alongside advances in MRI, there has been a significant increase in therapies to reduce inflammatory disease activity and consequent disability accumulation (disease-modifying treatments), from earlier studies on interferons, most prominently to a range of anti-CD20 treatments. Although effective, a significant proportion of people with MS continue to experience clinical or radiological disease activity and/or progressive accumulation of disability and none of the presently available treatments convincingly reverse established damage or disability [35,36]. The worldwide prevalence of MS is steadily increasing [37], meaning that the development of new treatments remains a key priority.

## 2. Multiple sclerosis subtypes and treatments

The following four well-established subtypes of MS exist based on clinical understanding: (i) clinically isolated syndrome (CIS), (ii), relapsing–remitting MS (RRMS), (iii) primary progressive MS (PPMS), and (iv) secondary progressive MS (SPMS) [38]. These groups are primarily defined by their patterns of clinical disease activity (clinical relapses and accumulation of disability), supported by MRI. A further less well-established category exists known as radiologically isolated syndrome, in which patients are diagnosed (through MRI) with pre-symptomatic demyelination, with radiology consistent with MS. The clinical significance and management of this category of patient are the subject of debate and ongoing research [39].

### 2.1. Clinically isolated syndrome

**Figure 3.**
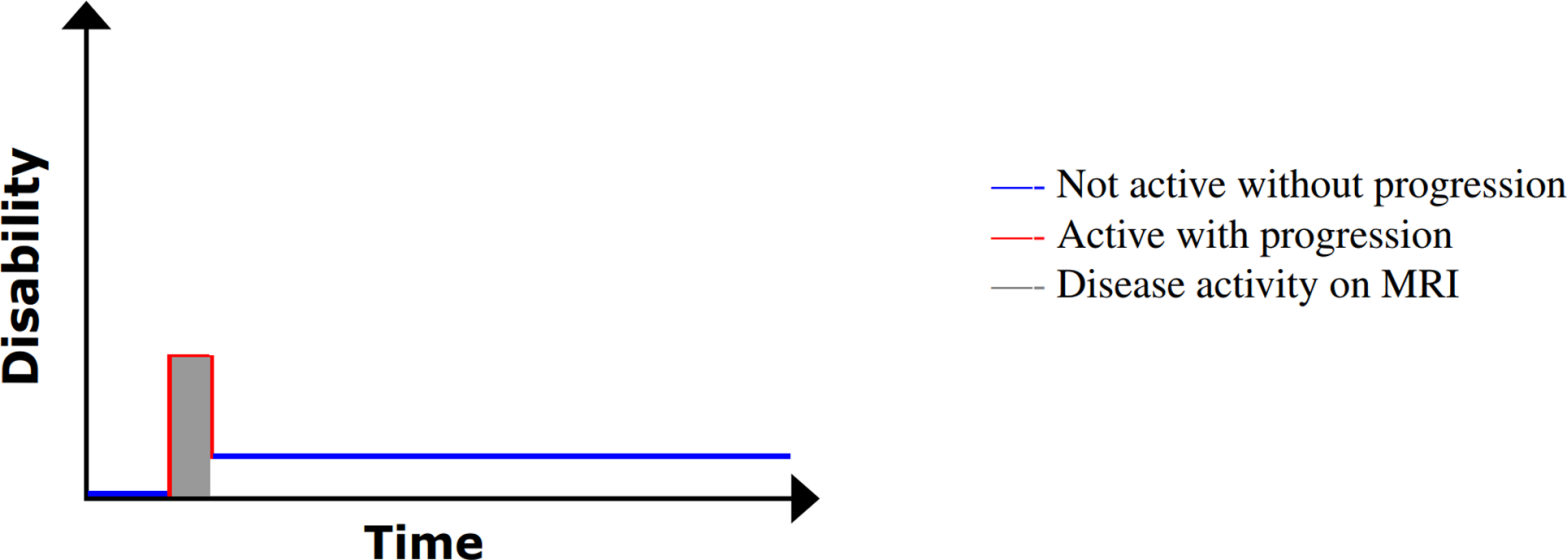
An illustration of disability progression with time for CIS. Image adapted from My-MS.org [42].

The CIS diagnosis involves a single clinical event, usually the result of a focal inflammatory demyelinating lesion within CNS with resultant symptoms consistent with MS [40]. By definition, the episode must be 24 h in duration or greater and without evidence for an alternative trigger, such as infection [41]. The inflammatory damage typically manifests itself in a spatially isolated area, namely a single lesion. This is illustrated in figure 3, in which the red line forming a raised peak is representative of a clinical attack that later subsides, the residual long-term effect of which is increased disability (the level of baseline disability does not return to a baseline level following the event).

The clinical presentation of the CIS can include optic neuritis or transverse myelitis, or less specific symptoms such as fatigue or cognitive issues [40]. With respect to treatment, CIS often resolves itself without the need to administer medication; however, corticosteroids such as methylprednisolone are often used to reduce inflammation and speed up patient recovery [43]. It should be noted, however, that steroid treatment does not impact either the likelihood of subsequent relapses or general long-term prognosis, i.e. is not considered disease modifying [44]. CIS can be a single event in the life of a person with no recurrence, but if a person experiences two or more episodes, this would confirm disease chronicity and their diagnosis should be changed to ‘clinically definite’ MS [45]. Even in the absence of clinically evident symptoms or signs, if an individual with CIS is monitored radiologically (via MRI) over time and there is evidence of MRI change (for example, the appearance of new MRI lesions), this again suggests disease chronicity and that a change in diagnosis to MS should be made accordingly.

### 2.2. Relapsing–remitting multiple sclerosis

**Figure 4.**
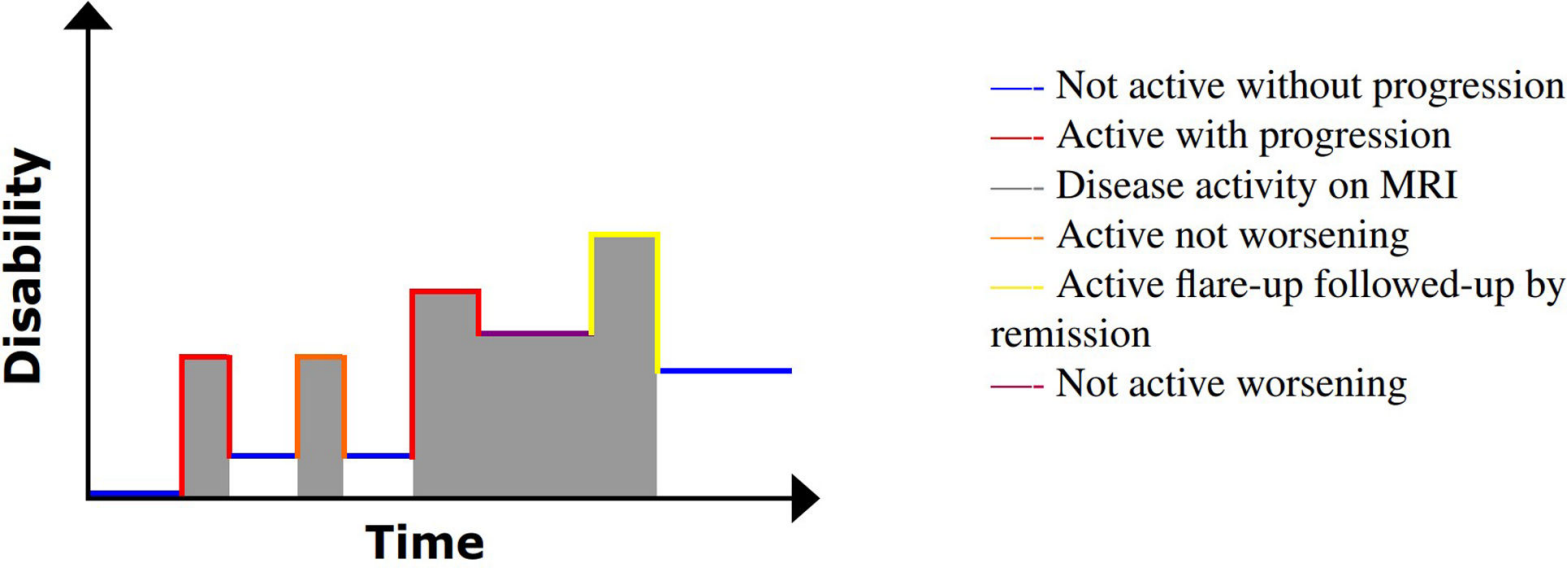
An illustration of disability progression with time for RRMS. Image adapted from My-MS.org [42].

The most common form of MS is RRMS, with approximately 85% of all MS cases being diagnosed with this subtype [46,47] and often following an episode previously labelled as CIS (the first clinical event). RRMS is characterized by at least two separate bouts of inflammation in the brain [48], which evolve over 48−72 h, the symptoms of which can last anywhere between two and eight weeks before subsiding. Sometimes an affected patient will fully recover from an episode, although it is more common for recovery to be only partial [49], so that the level of any resulting disability will fluctuate and even accumulate over time. This can be seen in figure 4, in which disability fluctuates with time and clinical attacks are episodic. The greater the number of relapses, the greater the likelihood there is of permanent disability [50]. Disease-modifying therapies (DMTs) aim to reduce inflammation, reduce clinical relapses and reduce the resultant accumulation of disability [51].

As with CIS, corticosteroids are often prescribed to reduce acute tissue inflammation [52], but steroid-related side effects are significant including insomnia, increased blood pressure, loss of muscle, weight gain, diabetes, cataracts, mood disorders and osteoporosis [44,53]. As a result, steroids are typically administered as a short ‘pulse’ for 3–5 days.

There are now multiple DMTs that can be used in RRMS treatment [54]. Depending on the specific type, DMT can be administered as oral medication, subcutaneous or intramuscular injection or intravenous infusions. The best-established therapies were preparations of interferon-β with demonstrable effects on clinical and radiological MS activity, acting through ill-defined mechanisms. Interferons are naturally occurring proteins, divided into the following three subtypes: α, β and γ [55]. It is interferon-β that is used to treat MS [54,56,57]. Two varieties exist, β−1a and β−1b, produced in either bacterial or mammalian cell cultures [58].

Over the subsequent years, the development of biological (infused antibodies) agents and small molecules have revolutionized the clinical management of MS. One is natalizumab (branded as Tysabri), a recombinant antibody that binds to the adhesion molecule (α4-integrin) on white blood cells [59], reducing inflammatory cell transmigration across the BBB, resulting in reduced radiological and clinical disease activity.

### 2.3. Secondary progressive multiple sclerosis

**Figure 5.**
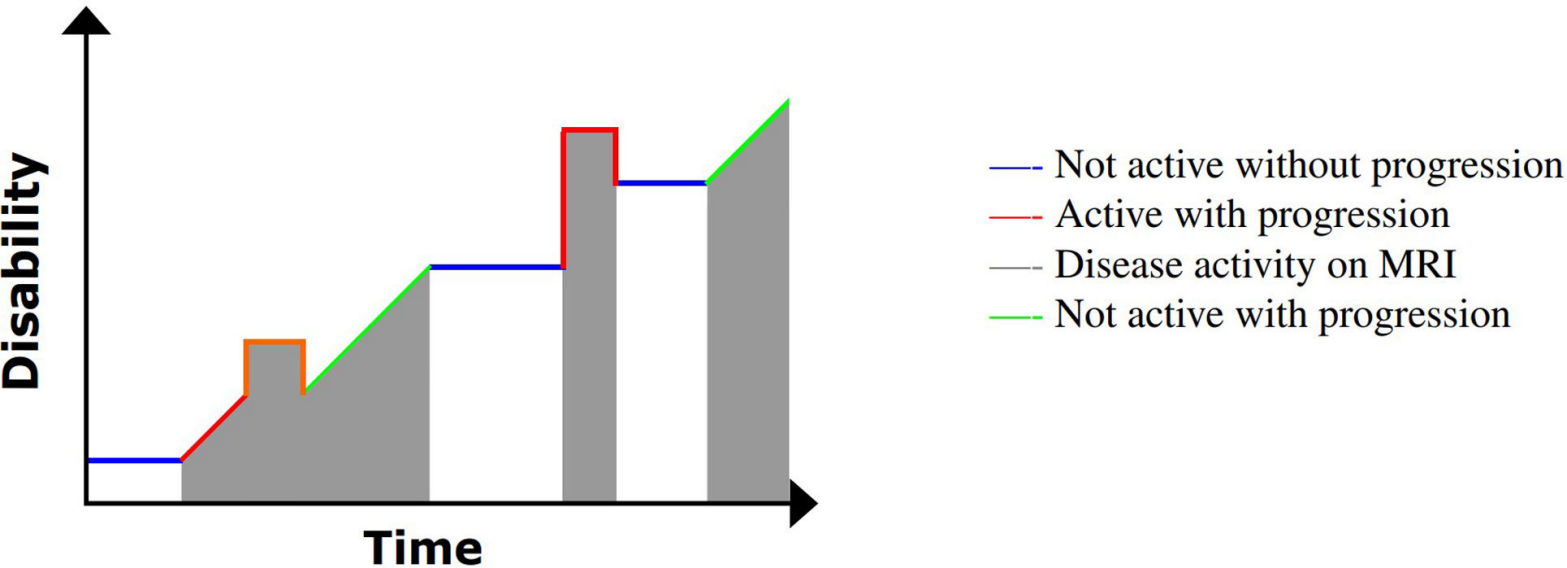
An illustration of disability progression with time for SPMS. Image adapted from My-MS.org [42].

A further stage in the clinical evolution of MS includes a more prominent role for non-relapse-mediated disease progression—SPMS, in which the relapse–remission cycle is less clinically and radiologically evident [60,61], typically evolving into a more continuous accumulation of disability without clear evidence of recovery from disability. This is demonstrated in figure 5; clinical attacks do occur, but worsening in disability can occur without an inflammatory exacerbation.

The term ‘progression’ is defined as the continual deterioration of neurological symptoms (or signs) for at least six months [38,61]. The condition is often retrospectively diagnosed [60]. SPMS is more challenging to treat than RRMS as DMTs have less well-defined effects on progression [62]. For non-active SPMS, the current standard treatment is primary through physiotherapy to improve strength, balance and mobility and occupational therapy for energy conservation and speech and language therapy to maintain bulbar function [63].

### 2.4. Primary progressive multiple sclerosis

**Figure 6.**
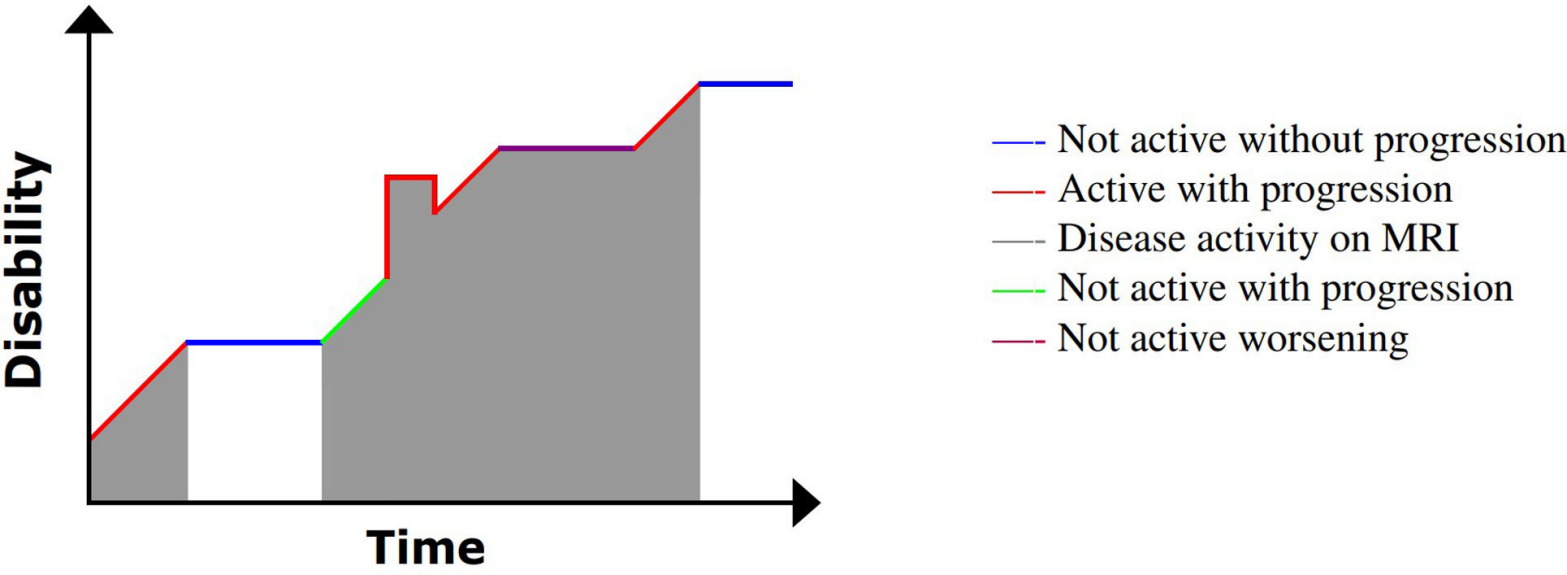
An illustration of disability progression with time for PPMS. Image adapted from My-MS.org [42].

The final MS subtype, PPMS, is characterized from the stage of diagnosis with continuous accumulation of disability (frequently mobility) and no sustained periods of remission [64]. This continuous disability progression is displayed in figure 6.

Between 10 and 15% of MS patients are diagnosed with PPMS [65]. There are several characteristics that distinguish it from other MS subtypes: lesions are more frequently located in the spinal cord and surrounding white matter, whereas cerebral lesions are more limited as compared to other MS subtypes [66]. Another feature that differentiates PPMS is that while every other subtype of MS disproportionately affects women, the gender distribution of PPMS is approximately equal [64]. In a similar manner to SPMS, DMTs for PPMS are more limited. At the time of writing, the only approved treatment for PPMS is the anti-CD20 antibody treatment ocrelizumab (also licensed for the treatment of RRMS) [35,67]. Although effective, it is, in essence, an anti-inflammatory, and like other DMTs, its impact is reduced in the latter stages of the disease when inflammatory activity is less prominent. Similar to SPMS, the mainstays of treatment are physiotherapy, occupational therapy and speech–language therapy, all of which are aimed at mitigating against the functional decline caused by the disease [63].

## 3. Regional lesion and atrophy progression in multiple sclerosis

**Figure 7.**
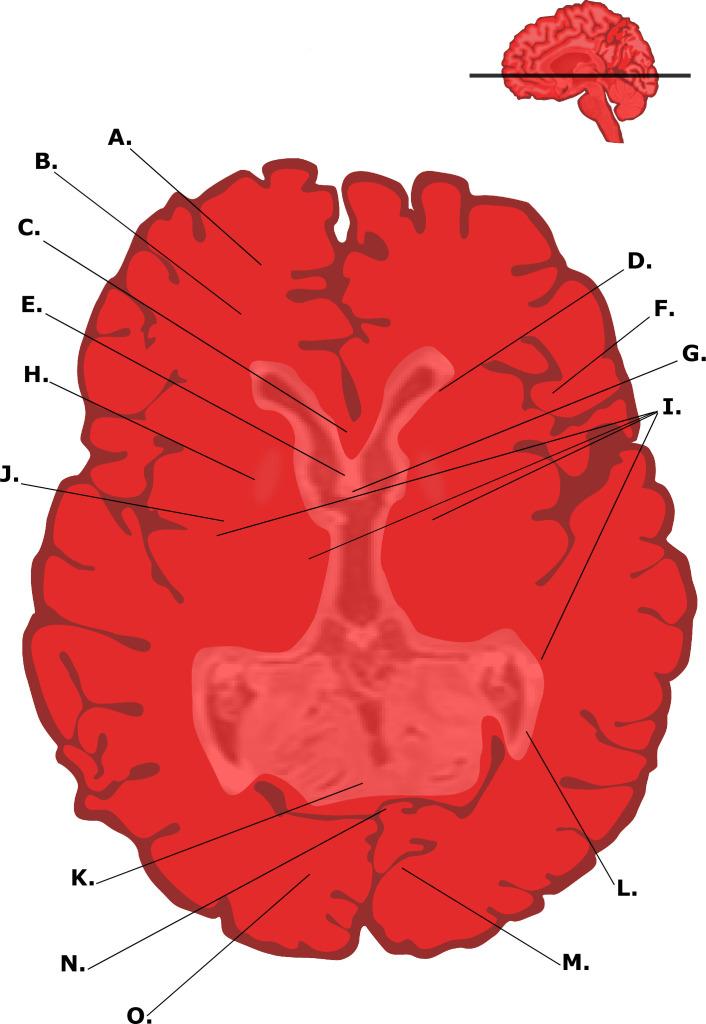
A labelled graphical illustration of a brain slice of an MS patient in the horizontal plane, where pale white spots are indicative of lesion location. A, frontal lobe; B, cerebral white matter; C, corpus callosum genu; D, lateral ventricle, anterior horn; E, septum pellucidum; F, cerebral grey matter; G, third ventricle; H, globus pallidus; I, white matter lesions; J, putamen; K, corpus callosum trunk; L, lateral ventricle, posterior horn*;* M, longitudinal cerebral fissure; N, posterior forceps; O, occipital lobe (original image).

The brain primarily comprises white and grey matter, which are both essential to healthy body functions, with damage to either causing serious negative consequences to a person. These tissue types are affected by MS differently, and understanding the pathogenesis with respect to both tissue types can offer a more complete insight into MS disease progression. A labelled brain slice of an MS patient in the horizontal plane is presented in figure 7, illustrating the distribution of white and grey matter, lesions and important areas of the brain.

### 3.1. White matter

In general, the damage inflicted on MS patients is most radiologically evident where white matter predominates. White matter is primarily located in the innermost layer of the cortex but also encases the spinal cord. Its constituents include glia such as oligodendrocytes and fibrous astrocytes, and it is light in colour due to its high myelin content. In the early stages of the disease, when relapses are most frequent [68], tissue damage is concentrated in the deeper regions of the brain such as the subarachnoid space and cortex, where white matter is prevalent [69,70]. Focal confluent plaques are common in the white matter of the periventricular region of the brain [13,68]. It has been reported that in these plaques inflammation is more prominent in earlier stages of their formation [68,71]. Their size and frequency increase with disease progression [72]. Microscopically, there is damage to the glia, the type of cells that help with neuronal protection and maintenance, with degeneration of myelin and loss of oligodendrocytes [68]. Demyelination usually occurs where myelin is in close proximity to activated macrophages or microglia [28]. This process most obviously manifests itself radiologically with the emergence of ‘Dawson’s fingers’ [73], appearing as vividly clear areas of demyelination on MRI scans. As mentioned in previous sections, some repair of myelin can occur during the phase of demyelination, which may correlate with clinical improvement as seen in RRMS. Astrocytic scarring develops during the healing process where any demyelination has occurred, creating a barrier between healthy and damaged or dead tissue and forming lesions [74,75]. Although lesion demarcation is a defence mechanism against uncontrolled CNS inflammation, it similarly impairs post-inflammatory tissue regeneration, likely contributing to tissue scarring and disability accumulation [75].

Active white matter lesions are most common during the early phase of the disease, becoming progressively less common as the disease develops as it transforms into the progressive phase [13]. By contrast, diffuse changes are minimal in normal-appearing white matter (NAWM) but increase as MS reaches a progressive stage [72]. Oxidative injury also occurs in the white matter, even in the early stages of MS [76]. In response to inflammation and the production of oxygen radicals, cellular damage occurs [68], specifically to the neuronal mitochondria, which results in virtual hypoxia: an energy deficiency leading to ionic instability in the damaged cells [13]. The reason for the prominence of this phenomenon as compared to other neuroinflammatory diseases is unknown, but as a consequence, respiratory deficiency in cells correlates with disease duration (and patient age) [68]. The impact of this form of damage becomes more important with disease duration and significantly contributes to oxidative tissue injury. In addition, during the later stages of the disease, the loss of oligodendrocytes becomes increasingly widespread, removing the primary mechanism of repair [77]. This, combined with axon loss, impairs tissue regeneration and repair. In terms of leukocortical lesions, present in both white and grey matter, remyelination tends to be less robust in the white matter than in grey [78,79], possibly due to differences in macroglial populations. Remyelination rates in white matter alone can also be dependent on the affected brain region. Subcortical white matter lesions, for example, are more likely to remyelinate than those situated in the periventricular regions [68,80]. Inflammation in white matter during the progressive phase of MS is similar to age-matched controls [68], suggesting that neurodegenerative processes are dominant in the latter stages of the disease [81]. In a similar manner, damage to the BBB progresses over time; when it becomes sufficiently severe, the level of permeability reaches a nadir such that diffuse injury in the NAWM begins to occur, potentially contributing to brain atrophy [68].

### 3.2. Grey matter

MS was primarily considered to be a white matter demyelinating disease, but radiological and histological advances over the past 20 years have confirmed that all cerebral tissue types are affected. Grey matter, from disease outset, is significantly impacted [73], but visualization and analysis of the resulting damage have, up until recently, been difficult [82]. As white matter demyelination (readily demonstrable on MRI) does not correlate well with many clinical symptoms (particularly cognitive), research into the drivers and consequences of grey matter injury remains a priority [83]. One key example is the strong correlation between cortical damage (for example, seen as atrophy in MRI) with cognitive deficits [84]. The fundamental pathological difference between white and grey matter lesions is that the former are usually associated with inflammation caused by leukocyte penetration of the BBB, while relatively few leukocytes are found in and around the latter [85]. Axonal and synaptic damage are characteristic of grey matter lesions [86,87]. There is significant variation in cortical demyelination in the early stages of the disease to that of later chronic MS [69]. Large quantities of cortical demyelination are characteristic of MS [88] and tend to be located in the sulci [89] and are associated with localized meningeal inflammation [68,90]. Although present in RRMS, cortical lesions become far more numerous in the progressive stages of MS, correlating with an increase in diffuse microglia reactions in the meninges and perivascular white matter [72]. It has been suggested that sodium channel damage and virtual hypoxia due to mitochondrial damage act as catalysts for grey matter degeneration [91]. It has also been reported that neurons with damaged axons require more oxygen to function, thus further straining oxygen supply and leaving them susceptible to further damage [92].

Grey matter damage is macroscopically characterized by cortical thinning—the shrinking of the cortex, which gradually increases with disease duration [82]—and does not seem to significantly vary with the course of the disease [93]. This phenomenon is largely confined to the frontal and temporal regions of the brain [82,94]. During the CIS and relapsing–remitting phases of relapsing MS, atrophic damage was first observed in the posterior cingulate cortex and precuneus, which was then seen to spread to the middle cingulate cortex, brainstem and thalamus [95]. Progression of atrophy in people with PPMS shares some common features with those suffering from CIS and RRMS but with some differences. The thalamus, cuneus, precuneus and pallidum were the first regions affected, with brainstem and posterior cingulate cortex injury subsequently occurring [96]. While the cerebellum, caudate and putamen appear to atrophy in the early stages of disease progression in the relapsing form, they become atrophic later in PPMS [95]. It is clear, however, that irrespective of the course of the disease, damage to regions of grey matter across the brain is unevenly distributed [97] and that damage does not develop in a stochastic manner [95,98].

### 3.3. Lesions

Lesions can be categorized by anatomical location, according to their visual appearance on MRI or inflammatory activity [20,99,100]. These classifications help in understanding the nature and implications of the lesions observed. The anatomical locations are as follows: periventricular (relating to lesions surrounding the ventricles), cortical/juxtacortical (lesions in and around the cortex), infratentorial lesions (pertaining to the cerebellum and brainstem) and those affecting the spinal cord and the optic nerves [99,101], with the latter often acting as an early indicator of MS [102]. Cortical lesions can be further divided into the following four categories: type I—leukocortical lesions, affecting cortical grey matter and juxtacortical white matter [103]; type II—perivenous intracortical lesions, affecting exclusively grey matter, which are usually small in size; type III—subpial lesions, which occur in the white matter on the brain surface, protruding inwards; and finally type IV—which occur throughout the grey matter within the cortex [20].

In terms of visual intensity, lesions can be divided into the following two categories: T2-weighted hyperintense lesions and T1-weighted hypointense lesions. T2 lesions appear brighter than surrounding white matter on T2, fluid-attenuated inversion recovery (FLAIR) and proton density (PD) scans (hyperintense). This relationship is inverted on T1 scans, in which lesions are either darker or indistinguishable from surrounding tissue. T1 lesions can be further divided into two subcategories: those that enhance when gadolinium contrast agent is administered (5 min prior to a T1 scan) and those that do not. The former, known as both gadolinium-enhancing lesions and paramagnetic lesions, indicate localized increased permeability (breakdown) of the BBB and the presence of current or recent inflammatory activity [104]. Without the injection of contrast agent, these lesions can be more easily missed by a radiologist or clinician. Other features may include ‘black holes’, focal areas of T1 hypointensity images. These do not enhance after a gadolinium injection and are thought to indicate foci of severe CNS damage relative to other lesion types [105] with permanent axonal loss [104]. Other non-enhancing lesions can occur, and in contrast to black hole lesions, they appear bright on T2 scans but of a similar intensity to surrounding tissue on T1 scans. They are typically chronic but represent less severe damage [106].

Lesions often develop and are capable of both enlarging and shrinking. This behaviour is associated with the level of cerebral inflammation [13]. As such, lesions can be categorized based on the level of inflammatory activity, which is typically related to the age of the lesion [74]. The categories are as follows: acute or active lesions, focal lesions, inactive lesions, chronic lesions, diffuse lesions and atrophic lesions. Newly formed lesions are, by definition, active and indicate both recent inflammation and demyelination [13]. At the cellular level, they contain macrophages and microglia throughout the lesion. Focal lesions can develop from active lesions and appear sharply demarcated, suggesting localized inflammation [74]. These lesions are indicative of more severe damage, including axonal loss, and can ultimately develop into black hole lesions. Inactive lesions manifest after the demyelination process in an area of lesion tissue has been completed. They have a hypocellular centre, with microglia limited to the periphery of the lesion [74]. While chronic lesions can remain latent, inflammatory activity can resume, potentially leading to further demyelination, lesion enlargement and atrophy. Chronic lesions in MS include several types, each with distinct characteristics. Slowly expanding lesions (or smouldering lesions) gradually enlarge due to low-level inflammation and are typically linked to degenerative processes [107]. These lesions often appear hyperintense on T2-weighted images. Black hole lesions (previously described) may also become chronic, with intermittent inflammation contributing to their progression [106]. Additionally, diffuse lesions (sometimes referred to as diffuse white matter injury) [72] are chronic but do not directly arise from other chronic lesions. Instead, they are associated with general cerebral inflammation, affecting larger regions of white matter. This diffuse injury tends to be exacerbated by bouts of inflammation (relapse and remission) [108]. Unlike active lesions, diffuse lesions do not have distinct borders [109]. Additionally, diffuse lesions can be linked to atrophic damage and overall brain volume loss [110]. Atrophy is a consequence of disease progression and cumulative damage.

## 4. Clinical disability metrics

There are a number of addition key terms, criteria and definitions used in MS. The McDonald criteria provide a consensus definition of the disease, with a particular focus on clinical and radiological (MRI) diagnosis, with updates over time, based on evolving research. Following diagnosis, quantification of MS-related disability over times is key, most typically using the expanded disability status scale (EDSS) and, to a lesser extent, the multiple sclerosis functional composite (MSFC).

### 4.1. McDonald criteria

Currently, the most commonly accepted formal diagnostic criteria for MS are the McDonald criteria, which replaced the Poser criteria in 2001 [111]. The pre-requisites for an MS diagnosis should include two clinical attacks, alongside the presence of at least a single inflammatory (active) lesion, MRI should be the imaging method of choice to demonstrate the latter. A number of revisions have subsequently amended, expanded and calibrated earlier definitions [100,101,112].

### 4.2. Expanded disability status scale

For practical purposes, the clinical definition of MS is based on the EDSS developed by Kurtzke [113]. A numerical scale of 0−10 is used and increases in increments of 0.5 to indicate the level of disability of a patient. This measure is subject to criticism, with relative lack of sensitivity, particularly in the latter stages of disability, where the EDSS is primarily dependent on mobility [114]. The EDSS has advantages of moderate simplicity, reasonable reproducibility and is well established and therefore remains a key element of MS clinical research, particularly clinical trials.

### 4.3. Multiple sclerosis functional composite

Due to the limitations of EDSS, an alternative metric known as the MSFC was devised [115,116]. It is arguably a more comprehensive measure of disability and attempts to improve on the EDSS. MSFC comprises three subsidiary metrics: a timed 25-foot walk (T25FT), assessing lower limb mobility over limited distance; a 9-hole peg test (9HPT), assessing upper limb mobility using a breadboard test (performed at least twice, once with each hand); and a paced auditory serial addition test (PASAT), a cognitive test assessing mental processing power by getting a patient to add up a series of 60 single digit numbers read out at 2 or 3 s intervals. The results of the T25FT and 9HPT tests are given in units of time, whereas the PASAT score is the number of correct answers given. Results are subsequently converted into generic metrics known as *z* scores [117]. These *z* scores are based on the test results of a patient and the average results of a healthy control population. The lower the MSFC score, the greater the disability, negatively correlating with EDSS [118], the scores of which increase with disability.

## 5. Computational methods applied to multiple sclerosis using magnetic resonance imaging

Fundamental questions surrounding the nature of MS remain unresolved, notably its precipitating cause, and optimal choice of treatment and management following the inflammatory phase. Moreover, other areas such as recognition of early disease and manual lesion identification can often be difficult and sometimes time-consuming for health professionals. Computational techniques may assist in overcoming these problems, unpicking the ambiguities of MS and improving diagnostic processes. At present, however, the application of computational methods applied to medical imaging within the areas of consideration remains relatively narrow. The general areas in which machine learning (ML), MS and MRI coalesce are in (i) diagnosis of MS and segmentation of lesions—a core aim of research upon which other areas are built, (ii) differentiating MS subtypes, (iii) prediction of disease progression, and (iv) distinguishing between MS and similarly presenting diseases.

### 5.1. Review methodology

The search strategy used for paper inclusion was fairly simple. The MEDLINE database was accessed through the PubMed search engine, and no date restrictions were implemented in the search process. Keywords used in the search were ‘Multiple Sclerosis’, ‘Machine Learning’ and ‘MRI’, revealing 171 technical studies published both in journals and at conferences and authored in English. Of these, the ones that did not include all aspects of the search were removed from the collection of studies considered. Furthermore, those studies comparing MS to its mimics were discarded from this review as it would require the inclusion of substantially more information unrelated to MS from a biological perspective, rendering this work unjustifiably lengthy. As a consequence, 25 studies were discarded. Currently, no reviews exist on the comparison of MS patient MRI to that of individuals with similarly presenting diseases using ML. There are studies that discuss MS and its mimics in general [119].

Recent systematic reviews around MS have focused on the diagnostic performance of AI in MS [120], ML algorithms used in MS analysis [121] and general AI application to MS [122,123]. There has also been work published on how AI can be applied to MS, but it is not always specific to MRI and lacks important contextual information and comparative details regarding the efficacy of segmentation techniques in published studies [124,125]. Additionally, a recent study paper has evaluated how ML can be applied to evaluate clinical measures in the context of MS progression [126]. However, there is a need to evaluate the application of ML more clearly as regards MS subtypes, which might provide valuable information on the fundamental biological causes of MS, the pathophysiological events occurring during the course of the disease (for example, mix between relapse activity and progression), with implications for clinical outcomes and treatment pathways, potentially highlighting gaps in knowledge. The future development of imaging-based technologies that exploit ML will depend on whether these clinical parameters are specifically addressed in ML datasets. Furthermore, this is the first study to compile all results from publicly available datasets (see appendix B), which enables the objective evaluation of the current state-of-the-art in ML techniques as applied to MRI datasets. This offers a benchmark for those wishing to directly compare the efficacy of any novel model using publicly accessible data.

### 5.2. Automated diagnosis and segmentation of multiple sclerosis lesions

The most rudimentary problems in the study of MS using computational techniques and MRI are its general diagnosis and the segmentation and quantification of brain tissue of MS patients. In general, the former involves distinguishing healthy brains from those of MS patients, while the latter focuses on the identification and segmentation of lesions or specific brain areas. In a clinical setting, lesion segmentation tends to be performed manually by medical professionals, which is time-consuming, often inexact and inconsistent. Automatic diagnosis and segmentation techniques, if perfected and adopted by hospitals, would free up the time for clinicians, allow for consistency of diagnosis to be applied to all patients (removing the necessity for manual delineation) and enable patients with the most aggressive and malignant disease courses to be rapidly prioritized by clinics. A variety of characteristics have been extracted in investigations assessing MS. These include lesion pattern, volume, shape and quantity.

#### 5.2.1. Objectives

From the inception of this field, pattern recognition has been pivotal to lesion assessment and behind most AI-based techniques that utilize MRI as an input [127], providing a basis from which lesions can be quantified [128]. Manual selection of likely lesion points was sometimes incorporated [129,130], acting as a basis from which analysis could be performed. The necessity for human input, however, was quickly rendered anachronistic, and end-to-end automatic segmentation techniques became the expected norm.

Automatic segmentation of lesions has been a hugely popular topic and is a wide-ranging field, with a plethora of studies conducted over the last 25 years [131–190]. These studies utilize a myriad of computational techniques and a variety of approaches, offering information about different aspects of MS. Since most readily available MRI modalities display white matter with the greatest clarity, segmentation of white matter lesions proved to be the easiest and thus most popular to investigate initially.

Segmentation of specific areas of the brain has also been a popular area for study in MS. Brain atrophy is a significant consequence of MS, with a reported rate of volume loss of between 0.5 and 1.35% per year [191–193]. Some studies aimed simply to distinguish between white matter, grey matter and cerebrospinal fluid (CSF) [164,182,184]. Other studies concentrated on the quantification of specific brain tissues like grey matter [194], white matter [181] and cortical thickness [195] for the purpose of monitoring cerebral atrophy or used cerebral spatial features [196] to gauge brain tissue degradation and deformation. Assessments of atrophy of specific areas of the brain, notably the thalamus [197] and the cerebellum [147], have been undertaken. General diagnosis of MS has also been explored [198–200]. These investigations are sometimes challenging because study participants tend to be comprising healthy controls and CIS sufferers or patients recently diagnosed with MS. The benefit of such studies is that they assess the possibility of a diagnosis of MS in individuals in whom clinicians have been unable to arrive at a satisfactory conclusion.

#### 5.2.2. Magnetic resonance imaging in lesion segmentation

T2 MRI was initially the standard sequence for identifying MS lesions [127,128,130,143,166]. The notable limitation of using T2 scans exclusively is that lesions appear indistinguishable from CSF [104], which is especially problematic when attempting to identify lesions around gyri and sulci, the latter of which is filled with CSF. Remedial measures implemented have been the inclusion of PD scans as a separate sequence [152,163,187,201] or combining the two sequences [151,202], to enhance lesion visibility. As the field has progressed, it has become standard practice to include multiple MRI sequences for each patient, maximizing the results produced by the methods implemented, with separate T1, T2 and FLAIR images [132,153,168,170,174,176]. A number of other imaging types have been introduced, including functional MRI [203] and diffusion tensor imaging (DTI) [204,205], but their utilization in studies of this nature has thus far been limited.

#### 5.2.3. Algorithms and methods

**Figure 8.**
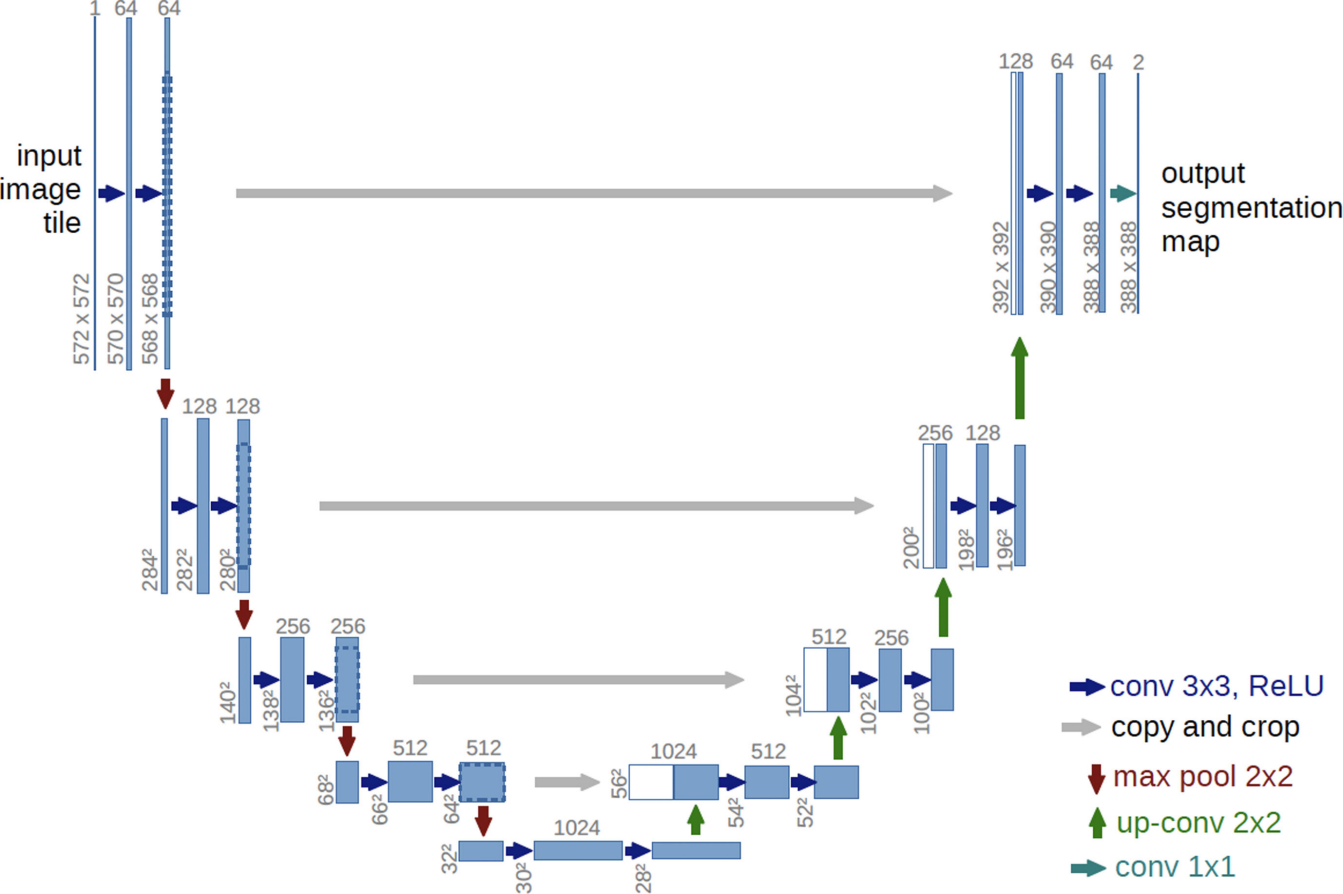
U-Net architecture: blue boxes represent multi-channel feature maps. The amount of channels is indicated by the number above the box. White boxes represent replicated feature maps. Arrows represent specific operations utilized [207].

A wide range of algorithms have been used in diagnosis and segmentation. Variations on fuzzy connectivity—a seed-growing method in which neighbouring pixels of a similar intensity are considered to be connected—were initially a popular method for segmentation in MS studies [130,162,165,166,169,173,177]. Supervised learning techniques, however, like support vector machines (SVM) [145,168,170,181,186,195,203,205,206] and K-nearest neighbours [144,164,178] soon became more popular owing to their lesser computational expense. In 2015, the field of biomedical segmentation underwent a revolution with the publication of the seminal paper ‘U-Net: convolutional networks for biomedical image segmentation’, the architecture of which can be seen in figure 8 [207].

Arguably, its main benefit has been its ability to produce reliable results from relatively little data input. In the International Symposium on Biomedical Imaging (ISBI) cell tracking challenge, it beat the next best method by over 30%. Convolution neural networks (CNNs) became widespread in medical segmentation, including automatic MS lesion delineation. While a number of unrelated CNN architectures have been used, V-net [139], GoogLeNet [133] and other 3D-CNN architectures [208], the vast majority credit the architecture proposed by [207] as their inspiration [132,134,135,146,148,150,152,180,182,185,188,209–212].

#### 5.2.4. Metrics

There are four general feature categories that can be segmented in brain MRI: grey matter, white matter, CSF and lesions. Lesions are the most difficult to segment as they are often small and have a heterogeneous distribution. Since no single metric encapsulates every pertinent piece of information necessary to fully assess an algorithm, multiple methods for gauging its efficacy for lesion detection and segmentation have been formulated.

The metrics conventionally used are the ones outlined and discussed in detail in appendix A: volume difference, sensitivity, specificity, positive predictive value and Dice score. At least one of these metrics acts as a measure to evaluate nearly all computational techniques involving the use of MRI discussed in subsequent sections. Refer back to this section for further information.

#### 5.2.5. Inferences and conclusions

The foundational work for automatic lesion segmentation was proposed by [127], in which computer-assisted methods were proposed and out-performed manual segmentation by 5%. The initial standard set for methods was to detect 100% of white matter lesions while avoiding false negative detection [128,162]. From early on, a variety of implementations had been reported that improved results, including varying window size in order to alter image scale [162] and the use of multidimensional feature spaces [164]. One of the major hurdles, as previously mentioned, was false lesion detection, not well accounted for by the sensitivity, specificity and accuracy metrics. Methods have been proposed to limit this phenomenon, but this has a tendency to compromise overall sensitivity [168,169]. Smaller lesions tend to be more difficult to detect [152], and it was reported in a study that results for lesions ≤20 µl in size scored 0.69/0.71/0.67 TPR/FPR/DSC only to improve to a near-perfect 0.99/0.02/0.91 when lesions only ≥500 µl were studied [182].

Some studies used solely T1 scans expressly for the purpose of segmenting black hole lesions [165,166]. This is a more challenging task due to there being a greater similarity in intensity between these and other surrounding brain tissue. Although some of these lesions are also visible in T2 scans, it was also suggested that there is the greater correlation between clinical disability scores and black holes than those found in T2 images [213], thus making them of greater importance. T2 lesion investigations are not, however, likely to be completely irrelevant as a later study found a linear relationship between grey matter volume and T2 lesion load [194].

In terms of cortical lesion detection, type I and type III lesions were detected with a high degree of accuracy, 83 and 70%, respectively [190]. Type II lesions (with the possible inclusion of type IV lesions), by contrast, proved more elusive (53%). This may be due to their smaller size and that they are predominantly located in the grey matter. Modalities outside of T1, T2 and FLAIR have also been investigated. Diffusion-weighted imaging, in particular DTI, utilizes the anisotropic water diffusion properties of tissue [104] and is known to yield information at an earlier point in time than other more commonly used MRI sequences. As automatic lesion delineation is more challenging when MS is in its prodromal stages [214], DTI could be helpful in earlier diagnosis. Commowick *et al.* [131] were the first to outline a framework by means of which this could be implemented in lesion detection [204]. It was found that DTI was capable of locating points of interest where lesions ultimately go on to manifest themselves even before their visible formation [215]. A further conclusion reached about imaging types is that with access available to multiple MRI sequences, the inclusion of all possible image types could be beneficial for training in order to maximize the information available to an algorithm. An investigation segmenting enhancing lesions [150] included five multispectral image sets and produced better results than when any single image set was used in isolation.

#### 5.2.6. Limitations

One of the most fundamental limitations in the studies has been data size; some studies are limited to relatively few study participants (five MS [128], two MS and two controls [216], 16 MS [181]). The validity of ML techniques and the success of the outcome are very much dependent on the quantity of available data. With as few as two patients in certain studies, the conclusions that can be drawn are inherently limited. Greater availability of public datasets for all imaging protocols would mitigate against this issue [188]. In the case of MS, this is especially problematic because of the heterogeneity of the condition in terms of the multiple pathological processes and lesion types [163]: a difficulty outlined in previous reviews [217,218] and papers [212]. If only one MS sub-diagnosis is included [196], or if there is a class imbalance [180,199,210], the ability of a method to generalize across all MS types is severely reduced. Furthermore, with a small dataset, it is unlikely that every disease permutation is accounted for which also has the potential to skew analysis in favour of a less commonly occurring subtype (if one is included). Therefore, in order to create a universal software program for clinical use, examples of every lesion variety and disease subtype must be included in the training data. The absence of even a single lesion type is likely to significantly impact the efficacy in terms of detection [175].

Regarding imaging itself, it is best that a variety of imaging protocols are utilized. In an early study, gadolinium-enhancing lesions were hypothesized to appear bright against NAWM in T2 images, but in reality, contrast was sometimes poor [161]. With poor contrast, the training process can be affected, leading to false-negative and false-positive lesion detection [162,167]. In another study, it was reported that chronic lesions were not detectable on FLAIR images owing to low contrast [168], whereas they were perceptible using T2-weighted images in other studies discussed. It has also been reported that smaller lesions [135,170] and limited lesion load [198] make accurate detection and segmentation difficult, especially in situations of low MRI contrast. For some lesion types, such as black holes [165,201], cortical and intercortical lesions [137,190,209], true prevalence is difficult to establish, even for some radiologists, especially when an unsuitable MRI sequence is utilized. All of these limitations illustrate the importance of using multiple imaging protocols to maximize the chance of lesion detection in studies in which a complete assessment of lesion activity is being attempted. It should be noted, however, that the algorithms used in a collection of studies using the publicly available Medical Image Computing and Computer-Assisted Interventions (MICCAI) 2016 dataset were sensitive to scanners and centres in which training data were not collected [131], suggesting that using a variety of scanners might not be of prime importance for engineering a generalizable software.

While datasets have expanded as the field has developed with studies including progressively larger cohorts, validation and ensuring a reliably delineated ground truth arguably remain the greatest limitations in this set of investigations [130,135,143,164,194,219]. Even in published competitions like that of Styner *et al.* [220], the reliability of the ground truth is questionable and has implications for the results of many other studies [141,144,154,157–159]. One publication associated with the most recently available MS MRI lesion dataset (MICCAI 2016 dataset) stated that there was significant discrepancy between the seven experts when defining ground truth for lesion location [131]. It is often difficult to enlist the time of expert radiologists or clinicians to perform ground truth segmentation, and some datasets use only one or two medical experts for this [176]. In an ideal scenario, generating ground truth should be done with the participation of as many experts as possible. Each segmentation performance should then be cross-referenced between themselves and averaged, with any anomalous outlying areas excluded, in order to obtain the most accurate reference image for algorithmic methods to be trained on or tested against.

### 5.3. Differentiating between multiple sclerosis subtypes and lesion types

Any diagnosis made should be as precise, or accurate, as possible. With MS, however, this is not a straightforward process because of the overlap that exists in the different subtypes. For example, it is still possible to experience a relapse after a diagnosis of SPMS has been made, and the EDSS scale, upon which conventional diagnosis is dependent, is itself, at least partially, based upon clinical features and self-assessment rather than purely objective measures. Furthermore, the varying presentations of MS lesions can also be categorized, which is useful as they are indicative of different types of damage, for example, enhancing lesions are associated with inflammation, diffusely abnormal white matter is linked to atrophy and black hole lesions are indicative of axonal damage [74,221]. There is always cross-over between MS subtypes and lesion types and lesions can be identified based on visual appearance, inflammatory activity and anatomical location—all of which can also overlap. Computational techniques can make use of objective lesion characteristics, such as volume, shape, visual intensity and location, and have been used to assess how closely lesion presentation reflects subtype diagnosis or lesion type to find a reliable empirical metric.

#### 5.3.1. Objectives

Various permutations of studies have been attempted, using either clinical subtypes or lesion characteristics as reference points. These have included several comparative investigations of MS subtypes. RRMS (chronic MS) comparisons against SPMS (acute MS) were and continue to be a popular area of research as the former tends to evolve into the latter [153,222–226]. Precision in determining the point in time when one subtype transforms into another is critically important for optimizing treatment because of changes in levels of inflammation, damage caused by demyelination and level of patient disability. Inclusion of other subtypes has been used in multi-classification studies, for example, separating the progressive subtypes into SPMS and PPMS [225], and multiple binary studies, comparing controls, CIS, RRMS, SPMS and PPMS patients in a single study [224]. Other standards used for making binary comparisons have included contrasting early-stage and late-stage MS, individuals with high and low lesion load and those with benign and malignant MS [227].

Linking demonstrable neurological deterioration to decline in motor functionality is the basis for the field of neuroscience. Thus, level of cognitive impairment, based on neuropsychological testing, has also been explored; the three classifications used are cognitively preserved, mildly cognitively impaired and cognitively impaired [228]. This study attempted to correlate demyelination and neural atrophy with physical and cognitive disability but excluded conventional MS subtype definitions.

As interest in the application of ML to medicine has grown, clinicians and radiologists have become more active in contributing to the supervised learning studies, leading to their increased recruitment to assist with providing more precise categorization of lesions, for example, in white matter, rim and intracortical lesions [229], and infratentorially versus supratentorially located lesions [230]. Other investigations have been carried out that directly build upon an aforementioned multi-classification study [225], but that use the following feature definitions: persistent black holes, persistent grey holes, acute black holes, acute grey holes, non-black or grey holes and NAWM [231]. Distinguishing between gadolinium-enhancing (paramagnetic) lesions, which become more visible after administering gadolinium contrast, and those that do not (non-enhancing lesions) has also been undertaken using artificial neural networks [222,232]. More recently, distinction between different types of cerebral tissue damage has been examined, diffusely abnormal white matter indicating widespread myelin damage and focal lesions [221], the difference of which may indicate severity of the MS and inform treatment decisions. Arguably, however, the most intriguing approach requires the least clinical contribution. It has become possible to sort MRI datasets based on lesion appearance using unsupervised learning. This was initially carried out using exclusively CIS patients [233], but this was later extended to use all subtypes [234]. It is possible that ultimately avoidance of the use of subjective metrics will prove fruitful and yield greater insight into as-yet unexplored areas of MS.

#### 5.3.2. Magnetic resonance imaging in lesion and subtype identification

The MRI sequences used in these studies can be divided into three types: T1, T2 and FLAIR. T1 was initially the most popular MRI sequence used as data for computational techniques [221,222,227,228], with administration of gadolinium in some instances to enhance image quality and to help distinguish between paramagnetic and inactive lesions [222,232], highlighting active inflammation. Little justification has been given in any of these investigations for the choice of T1 over the other options, but it is known that lesions best demarcated using T1 MRI correlate most strongly with physical disability [235], so this may have been one consideration. Alternative enhancement agents have also been investigated, notably ultrasmall superparamagnetic iron oxide (UPSIO) [233], with which T1, T2 and FLAIR MRI were explored in some capacity. The UPSIO highlighted a separate collection of lesions to those highlighted with gadolinium. These lesions were indicative of a more aggressive disease course—thereby emphasizing the usefulness of UPSIO. In practice, the choice of MRI is more useful for highlighting inflammation activity and lesion type as opposed to the MS subtype. It was also demonstrated that T2 MRI highlighted a greater number of lesions making it beneficial for finding points from which fresh areas of demyelination could develop, highlighting both diffuse and focal damage [221,233]. Generally, more recent papers tend to use at least two types of MRI: FLAIR and T1 [223,224,230], T1 and T2 [229,231], FLAIR, DTI and T2 [225] as well as all three [153,234]. The greatest benefits of using multiple MRI sequences are that it increases the dataset size as well as also presenting the neural network with essentially new information, thereby making it more effective in improving subtype identification [236].

#### 5.3.3. Metrics

In studies using computational techniques for examining MS subtypes or lesion subcategories, the two simplest metrics for assessment were number of lesions present and the lesion volume. By simply counting pixels or voxels making up an image and then utilizing a method to count objects of interest, both metrics can be calculated. Since lesions can, however, develop in areas of the brain which if damaged do not necessarily lead to disability, they are limited metrics [222]. Consequently, most papers, even from the inception of the field, concentrate on lesion topology [222,223,228,230,233]. Lesion volume [221,223,234] and number of lesions [224], however, have been used in numerous papers in combination with other metrics because it is known that physical damage in specific areas often results in disability, and with this being the case, lesion location has been used as a metric to distinguish one subtype from another [229,231,236]. As technology has improved and MS has been increasingly understood, more focused studies have been undertaken. The markedly more difficult task of investigating MS lesion manifestation in grey matter has also been conducted [227]. One particular study sought to establish which available metrics offered the best results for training an algorithm to distinguish between subtypes. All data were temporally longitudinal and included lesion characteristics and clinical and metabolic features [224]. As the field progresses, more specific data types like nuclear magnetic resonance spectroscopy signals are likely to be increasingly explored [226], the most immediate advantage of which is that they are computationally inexpensive to process relative to those from MRI images.

#### 5.3.4. Algorithms and methods

A number of models have been used to differentiate MS lesion types and subtypes from each other. The first paper published on the topic used a combination of fuzzy logic (fuzzy connectivity) and input from human experts who selected the location of lesions by direct inspection [222]. As the field has developed, human input, post-data pre-processing and model construction have become less of a requirement. A wide variety of supervised learning methods have been utilized to sort data labelled by clinical experts including SVMs [224,226,227], CNNs [153,221,236], MLPs/DNN [231], random forest algorithms [224], gradient boosting [229] and Bayesian neural networks [223]. Unsupervised clustering methods have also proved popular in categorizing lesions into groups with similar characteristics [233,234]. Supervised learning techniques tend to have the capability of being more accurate because they include a ground truth, but there is a danger that in the training process overfitting to a particular dataset can occur, the phenomenon in which an algorithm fits so well to a training set that it is unable to perform well on unseen data. This can be particularly problematic if a single scanner brand is used in generating a dataset or if one subtype is disproportionately represented. The main benefit of unsupervised over supervised learning is that less pre-processing is necessary for the former and so needing less human intervention. In this context, the use of clustering eliminates any conscious or unconscious human-generated bias and established preconceptions by only utilizing the tangible characteristics of the data available. Nevertheless, the importance of diagnoses and patient evaluations should not be minimized as they can assist in improving algorithmic outcomes. In one study, for example, in which a clustering algorithm was applied, it was demonstrated that inclusion of clinical data in addition to the clustering technique improved algorithm accuracy [234].

#### 5.3.5. Inferences and conclusions

Lesion volume was found to be statistically significantly larger in RRMS than in SPMS [222], and an inverse relationship was found to exist between both disease duration and lesion quantity in terms of both number and volume. There was, however, no demonstrable correlation found between lesion volume and EDSS [222,225] due to lesions manifesting themselves in silent regions of the brain, i.e. those not responsible for bodily functions, suggesting a need for a more nuanced approach. Supervised learning methods were able to effectively differentiate between MS data, based on a variety of criteria with considerable success, including early-stage MS and late-stage MS (85%), low and high lesion load (83%) and benign and malignant MS disease courses (77%) [227]. In terms of differentiating between conventionally accepted subtypes, identification rates ranged between 71% and approximately 96% [153,224,226]. It was found that metabolic data were a more reliable means than MRI for discerning the differences between RRMS and PPMS, whereas MRI was better for differentiating between RRMS and SPMS [153,224]. Some of the best results were achieved using a pre-processing technique known as class activation mapping (95.42%), but it should be emphasized that overfitting could have contributed to these results as relatively few patients participated, and only a single scanner was used in the study [153].

Other data types have also proved to be effective in determining MS subtype, including isotropic fraction (reflecting cellularity) and fibre fraction (representing apparent axonal density) [225]. Using a hybrid DNN–DBSI methodology, based on lesion appearance, a prediction accuracy of 93.4% was achieved [231]. Additionally, a link was found between MRI appearance and level of cognitive impairment based on a measure known as centrality [228], illustrating the efficacy of MRI for tracking MS development. Studies on anatomical location of lesions have also been conducted [229,236], and it was found to be easier to identify infratentorial lesions (87%) than the supratentorial ones (62%) [236]. In both papers that explored the use of unsupervised methods, three lesion categories were found that had no correlation to established subtypes [233,234], consistent with a paper previously discussed in the MS progression section [237]. When CIS was explored in isolation, two of the lesion categories found contained a greater proportion of hypointense lesions, indicative of greater disease activity and lesion load. The *p*-values of each group indicated that the reconception was statistically significant (0.0001, 0.0021 and 0.0192) [233]. In a study in which all MS subtypes were explored, they were characterized in greater detail as cortex-led, NAWM-led and lesion-led [234]. The ‘lesion-led phenotype’ had the highest probability of developing 24 week confirmed disability progression (CDP), and it was also the only one that showed any significant response to treatment. The results showed promise for assisting with the medical stratification of patients for the purposes of optimizing treatment based on predicted disease progression. The concordance metric produced for predicting CDP (after a 24 week period), however, was not entirely favourable even with the inclusion of clinical information (0.63) [234]. Diffuse lesions have been found to be particularly difficult to detect, with a Dice coefficient of only 0.49 achieved compared to 0.81 for focal lesions in the same study [221]. This is likely a consequence of the subtle appearance of this type of damage on MRI scans.

#### 5.3.6. Validation

Validation methods can be divided into two categories: human expert and computational. Some of the earlier studies employed clinicians to validate study results [222,223], but this process is both time-consuming and susceptible to human error. Computational methods have therefore been preferentially utilized to improve the quality of validation and to reduce the need for human input. Leave-one-out (or multiple data points) cross-validation methods became particularly popular for achieving this [224,227,229,234], in which an algorithm is trained using a dataset with one or more random pieces of data left out for testing. The process is then repeated over a defined number of epochs and with a different portion of the dataset being used for testing in each epoch. Other methods simply define the training, testing and/or validation datasets [153,231,236]. As in the case of the leave-one-out method, multiple epochs can be applied to improve the accuracy of an algorithm in the classification process but with the caution that this could lead to overfitting, in which case it may perform poorly on unseen testing data. Furthermore, if too many epochs are defined, the process can become prohibitively computationally expensive.

#### 5.3.7. Limitations

Early papers required the need for a human operator [222]. Most of these studies have their specific limitations, such as the need for a human operator in the earlier ones, but there are also universal problems that span almost the entire range. Of these, one of the most significant has been dataset size with only between 19 and 100 patients in most instances [153,222–227,229,231,233]. Only a few publications exceeded this number, notably 332 [228], 1800 [236] and 9390 [234] MS participants. The more data that are available, the more effectively an algorithm can be trained. The drawback of using fewer patients is that potentially it generates an insufficient quantity of data to create a method that effectively differentiates MS subtypes, especially if the data are demographically homogeneous, such as when they are taken from a similar age group, geographic location or use only one or a limited number of scanners. Quality of scanner used can also be limiting since lower frequency MRI reduces the detectable amount of feature detail [224]. It is also important to stress that although the use of high-quality images improves accuracy, there is little practical sense in mismatching the use of high-resolution equipment for training with images from lesser quality scanners. For this reason, it was necessary to exclude some data, in particularly from more up-to-date and sophisticated scanners, which generated sensitive metrics in order to avoid skewing data and clustering images based on quality rather than on visible features [234]. Discussion has also arisen over whether MS could be in fact a group of diseases with similar characteristics and manifestations rather than a single disease entity with divergent courses [238]. Research into understanding individual subtypes with their respective courses may also help shed light on answering this auxiliary question. A gold standard for identifying certain lesion types does not exist. For example, in the case of diffuse lesions—a newer subject of interest—there is no agreed-upon clinical standard for identifying diffusely abnormal white matter [221].

### 5.4. Predicting multiple sclerosis disease progression

Monitoring MS and its progress is of fundamental importance to guarantee that patients receive both optimum management and assessment of the efficacy of their treatment. As outlined, regional atrophy evolves over time, and the areas of the brain affected vary between patients. For example, the ventricular system is particularly affected with evolution of RRMS, whereas in progressive forms of disease the cortex is pre-disposed to this [193,239]. These appearances therefore govern the management that would need to be implemented subsequently.

#### 5.4.1. Objectives

Elliot *et al.* were the first to implement a technique using ML methods involving longitudinal data on MS patients to predict the likelihoods of new lesions appearing, enlargement of any existing lesions and lesion resolution [240]. Other studies exploring MS progression, based on lesion development and appearance, have used a variety of criteria including image intensity, deformation fields [241,242], lesion volume [243–245], lesion enlargement [236], lesion age [246] and total lesion load [244]. Exploration of the rate of deterioration of MS symptoms comparing algorithmic performance against that of clinicians has also been a central theme of studies [247,248]. Due to the availability of T2 MRI and because MS is considered to be primarily a demyelinating disease, attempts to correlate the progression of white matter demyelination to physical or cognitive disability have proven to be a common recurrent theme in studies [237,249–252].

Monitoring cerebral atrophy and alteration in brain structure have also been used as objectives in research to monitor disease progression. One of the earliest studies in this area aimed to correlate morphological changes in deformation fields with patterns in the spatial distribution [242], which are a direct consequence of morphological changes caused by atrophy and lesion development. The benefit of this approach lies in the fact that the further an MS brain structure diverges from the norm, the greater the prospect of disability. Understanding how changes in the brain affect disability is vitally important for improving understanding of progression in MS. Models have been developed to attempt to correlate changes in the entire brain with progression of physical disability [247,252–256], but other models created have concentrated on specific areas of the brain, for example, thalamus [257], corpus callosum [258] and cerebellum [259]. MS is a phasic condition in which either CIS develops into RRMS or PPMS and RRMS develop into SPMS. General investigations on MS progression have been conducted involving all subtypes [253], but RRMS has been the subtype most widely studied in relation to MS progression [232,237,240,246,251,259], most likely because it is the most common form of MS. Progression from CIS to RRMS has also been a particular area of focus [241,249,252,254,260–263], as not all patients diagnosed with CIS ultimately progress to RRMS. Having the ability to gauge the likelihood of this happening may ultimately yield aetiological understanding, and if fully comprehended, lead to clinical protocol that might minimize the chance of progression. These arguments can be made similarly for investigating RRMS to SPMS transition [245]. Research has also been conducted to identifying the most significant features required for diagnosis and, in so doing, reducing the need for data collection in future studies [254]. One technique, recursive feature elimination analysis, was implemented to explore the most indicative characteristics of subtype transition [262]. Experiments have also been undertaken to assess the impact of certain medications on MS progression. Both interferon users, relative to a control group [263], and ocrelizumab users, relative to interferon users [232], were analysed to assess their efficacy in curtailing the advancement of MS.

#### 5.4.2. Metrics

The most common way in which analysis is conducted in predicting MS progress is through use of two or more sets of serial scans. Normally, a group of patients, who fit certain criteria (e.g. time since diagnosis and MS subtype), are inducted into a study for whom various data types including MRI scans and clinical test scores are collected to establish a health baseline for each study participant. After a designated period of time, each patient is re-assessed at least once to gauge disease progression. These re-assessments can be carried out at either regular or irregular intervals and can incorporate further MRI scans or disability evaluations. In most cases, the health of each patient will have deteriorated illustrated by the metrics collected. Lesions may be seen to have visibly grown or diminished in size and changes in brain morphology may have also evolved on serial scanning. Changes in numerical data can be tracked from longitudinal MRI datasets such as lesion load, total number of lesions and lesion pattern. Lesion load has been factored into the analysis of many papers tracking MS progression [236,237,240,241,243,245,248,252,254–257,260–263]. This has enabled straightforward comparisons to be made at different points in time. MRI texture analysis has also been a popular method. This assesses the heterogeneity of an image through the use of statistical models [249,251], ultimately extracting information such as tissue abnormalities that may precede lesion manifestation associated with a specific condition. Brain atrophy, to which disability can be reliably correlated, has commonly been used as a metric [255,256,258,259,263], but this is difficult to assess because of the ‘reserve capacity’ of the human brain [264,265]. Much of the research available utilizes more than one predictor, in order to determine which of them is most prognostic of disability progression [255,262]. Level of disability can be gauged through a variety of clinical assessment tools of which the most commonly used in the investigations outlined is undoubtedly the EDSS [232,237,243,245–249,251–256,258,261–263]. Other metrics do exist and have been employed both instead of and in addition to EDSS: these include the MSFC [242,259], McDonald criteria [237,243,253,254,260] and symbol digit modalities test (SDMT) [247,252,258]. These alternatives allow other aspects of MS disability, especially cognitive impairment and upper body mobility, to be considered, which EDSS does not assess sufficiently [114].

#### 5.4.3. Duration of observation

Timespan of observation is a key consideration of temporal MS studies. MS is a disease that often lasts for decades. With the improvement of medical care, life expectancy of people with the disease has increased, and it is not uncommon to die from unrelated causes. In a 60-year Norwegian study, it was shown that MS patients have only a 7.1 year reduction in life expectancy over the general population [266]. As MS is often a near-enough lifelong condition, it is clearly favourable to monitor a patient as frequently as possible. On the whole, yearly clinical and/or radiological re-evaluation appears to be an optimal interval. This ensures that a study is cost-effective, with a single MRI brain scan costing approximately £200 (NHS) and $980 in the UK and USA, respectively. Investigation of specific subtypes may require more specific temporal parameters. For instance, CIS needs to be assessed as quickly as possible after diagnosis as a second relapse indicates a diagnosis of RRMS, and this can happen quickly. Several studies have shown that the average time between CIS onset and assessment was under two months [261,262]. Most studies based wholly on MRI include at least a single year of observation [236,237,240,241,250,251], but usually more [232,243,246–248,253,255,256,258,260,263], in which an initial scan and at least one other subsequent scan are collected per patient. Some studies, like the aforementioned ones focusing on CIS, used solely clinical assessment in follow-up appointments as opposed to MRI. A number of more general studies refrain from performing any subsequent MRI scans and base outcomes solely on clinical diagnoses made by medical practitioners. These include the MSFC [242], McDonald criteria [254], EDSS [245,257], fall status and atrophy damage [259] and SDMT [252]. Although this has practical benefits, the absence of follow-up scans limits the scope of the study by omitting important information that allows for the visualization of progression in terms of demyelination and atrophy.

#### 5.4.4. Algorithms and methods

A variety of algorithms have been used to study MS progression. SVMs have been particularly popular, especially when using data in addition to baseline MRI scans in supervised classification problems [237,245,248,249,251,261–263]. Similarly, random forest algorithms [245,246,252,260] and multi-layer perceptrons have been used for supervised classification problems [256]. Regression models have also been incorporated into studies [241,245,250], two of which incorporate SuBLIME [267], which involves probability assessment of voxels making up a lesion after implementation of pre-processing. CNNs have proved to be highly useful in visualizing the progression of MS on MRI scans, especially in terms of demyelination and/or brain atrophy [242,247,253–255], generating images as outputs. Again, U-Net is often cited as the main influence in architectural design [232,236,242,243,257,258].

#### 5.4.5. Inferences and conclusions

Initial studies demonstrated that it was possible to make predictions using MRI scans. With the benefit of amplitude modulation–frequency modulation to extract features, it was possible to determine the likelihood of an MS patient having an EDSS of greater than two with 86% precision [249]. Other early studies focused primarily on documenting methodology rather than on generating significant results and illustrated either single or multiple methods for making predictions using computational techniques based on MRI scanning [240,242,250]. Some of these were conducted around binary classification, comparing tissue types at different points in time. Classification results in this study exceeded 85% for all but one of the seven comparisons [251]. As regards predicting conversion from CIS to clinically definite MS, results varied between 70.4 and 92.9% [254,260–263]. Accuracy in results, based on an average taken between sensitivity and specificity, declined over time with results dropping in one study from 71.4% at the 1-year mark to 68% at the 3-year mark. Sensitivity decreased while specificity increased over this period [261]. Addition of combinations of various features including MRI lesion geometry, EDSS, SDMT, sex, demographic and clinical features improved prediction accuracy [247,261,263]. SVMs have been one of the most effective ML techniques for assessing MS disease progression. An *R*-value of 0.8 was generated predicting EDSS using multiple features [245]. Under certain circumstances, matching EDSS to disease subtype was inaccurate due to RRMS and SPMS patients, and SPMS and PPMS patients both having the potential to produce similar EDSS [253].

In another study, which made use of multiple variables as well as implementing class imbalance (weighting towards sensitivity), an accuracy of 86% was achieved, with T2 lesion volume ranked as one of the best indicators of MS progression alongside age [248]. The significance of T2 lesion load was consistent with findings from other studies [256]. Other informative metrics included lesion load in the main white matter tracts in the cerebellum [237], cerebellar volume [259], lesion patterns [252], cerebellar lesion patterns (88% chance of worsening) [255] and T1-unenhancing baseline lesions [232]. Area under the curve, based upon true positive rate (TPR) and true negative rate, scored between 0 (no measure of separability) and 1 (excellent measure of separability), is another metric that has been used to predict disease progression. It was found that a combination of features, including lesion patterns, proved to be effective in predicting worsening cognitive and functional status in MS patients with scores between 0.79 and 0.90 [252,256,268]. A key conclusion was that radiological features were more accurate classifiers in predicting disease progression than clinical ones [245,246,256]. Predictions were also made using Dice, for segmentation for which results ranged from 0.56 to 0.908 [232,241,243], with contrast-enhancing lesions achieving the best results [243]. Demonstration and quantification of cerebral atrophy, using MRI, has also been used as tool for exploring disease progression in MS. The feature can be used to calculate disability levels and to predict future disability and cognitive decline (*p* = 0.025) [257]. Corpus callosal atrophy was similarly shown to be able to predict disability based on EDSS up to 10 years into the future, with *p* < 0.001 produced [258].

#### 5.4.6. Limitations

The limitation of insufficient dataset size also applies to researching MS progression. Some studies have included as few as 20 patients [250]. The issue is compounded by MS having the potential to evolve in different ways. This unpredictability means that within a small sample, a certain subtype(s) may become either under- or over-represented skewing the results. This could especially be a problem in any generic study about MS. For example, a study conducted on transition of CIS to RRMS contained markedly few patients who did not progress to having a second relapse [263]. Furthermore, some papers treat MS as a homogeneous condition [253,254], despite there being numerous universally accepted pathogeneses, and that a number of recent computational studies have divided MS into three pathways based upon lesion pattern [233,234,237]. In studies concentrating on MS progression, study participants sometimes miss examinations, leaving gaps in datasets. For example, one study of 83 patients that used 455 scans was illustrative of this (as 455 is not equally divisible by 83) [253] and was common across relevant studies [240,261]. Data collection is often also taken at irregular intervals or not immediately after an incident of interest takes place. An issue unique to MS progression and deterioration is that some papers only include one MRI volume per patient, normally performed at the initial assessment, subsequently preferring to use clinical assessment metrics in isolation as a measure of disability [242,245,252,254,257–259], meaning that there is no way in which physical tissue damage can be correlated with disability. Additionally, the duration of monitoring is crucial. All papers cited described carrying out at least one follow-up assessment within a year of initial contact. Only five of over 30 of the papers discussed in this section involved patient data collection of at least 5 years [243,246,248,253,256]. MS is a disease that can debilitate an individual for decades, so it could be argued that tracking progression for only a single year is insufficient for gleaning the maximum amount of useful information.

Many studies do not include healthy control groups, which would be helpful to ensure that results are unique to MS [259]. This would help with overcoming some problems as one algorithm designed to estimate EDSS had trouble with predicting a score of zero [255]. EDSS is often used in studies either as a metric for disease progression or as an additional piece of data alongside MRI scanning to establish rate of deterioration. Nevertheless, EDSS does not take account of cognitive and visual decline, or upper limb disability [245,248,255,256], which also correlate to lesion development and brain atrophy. The MSFC, comprising mobility, cognitive and auditory tests, is arguably a more complete metric for gauging different aspects of disability [242,259].

Larger studies have tended to utilize data from multiple centres, either nationally or internationally. This has resulted in the use of a variety of scanners providing images of non-uniform resolution and size. In order to ensure uniform image characteristics when using different scanners, images produced by higher resolution scanners need to be reduced in quality, erasing useful information for neural networks to learn from in their training process [258]. The reduction in quality is especially important if an unsupervised ML technique is used to categorize scans, as it is feasible that it would use resolution as a criterion for sorting. Alternatively, it is possible to use images taken by the same scanner, a strategy which has been implemented in some investigations [232,241,243]. This does, however, limit its applicability to clinical and industrial work if access to the scanner that has been used to train the software is not available. Establishing ground truth for lesion location can also be problematic. For example, manual T2 lesion segmentation was reported as being only 62% [219], demonstrating the ongoing challenge of automatic lesion delineation. Moreover, the number of clinicians involved in manual segmentation upon which the ground truth is based was as few as two in some instances [232,247]. A further limitation in these studies is that there is not always record of whether a study participant has been medicated, making it difficult to establish the impact of treatment on data used in an experiment [195]. Treatment itself may influence the way in which MS manifests itself in individuals, as in lesion pattern, shape and development. Only two studies were found that specifically investigated the effect of treatment [232,263]. Without taking this variable into account, it is difficult to evaluate to what extent MS progression is governed by treatment and how much is part of the normal disease process. This reservation is further illustrated by the fact that the use of gadolinium as a contrast agent is thought to possibly underestimate lesion age [246].

## 6. Future developments

Larger datasets are essential to improve the predictive capacity of any algorithm [241,249,261]. Datasets have gradually increased in size over time, with pooling of national and international data and greater public availability. Some of the more recent studies utilize data from over 1000 patients [234], but many tend to not involve more than a single MRI per patient. Data augmentation methods and production of synthetic data both have the potential to imptove dataset size [254]. It would be a great technological advance to create a commercially viable product that incorporates data from multiple scanners to make a software package as universally applicable as possible [241,246,257]. Ultimately, one of the primary aims of research in this area is to produce functional software to aid clinicians in assessing and diagnosing patients by speeding up these processes and increasing diagnostic precision.

The inclusion of further features in analysis is likely to be beneficial, especially to determine which features are most influential in predicting disease progression [256,260,261]. Some of the features proposed include grey matter atrophy, intrathecal synthesis of oligoclonal bands and genomic data [261,262], as well as consideration of smaller subregions of tissue within the brain in isolation, such as dividing the cerebellum into subregions to better assess the tissue damage–disability relationship [259]. In relation to predicting progression, extending the timeframe for monitoring patients [237] and increasing the number of data points within the assessment period [253] have been proposed to obtain a fuller picture of MS pathogenesis. There is a strong argument for using MSFC as a disability metric as opposed to EDSS, as it covers different aspects of disability more comprehensively [237,242]. Encompassing multiple MRI protocols has been recommended because, as previously mentioned, certain lesions are more easily discernible, depending on the relaxation time used to generate the MRI image [232,252,256].

## 7. Conclusions

This review gives a comprehensive assessment and overview of the application of ML techniques to MRI scans performed on MS patients. Various aspects of MS have been described and discussed, including pathology, history, subtypes, white and grey matter damage, lesion development, clinical disability metrics and publicly available MRI datasets. The principal sections of the review explore three ways in which ML techniques have been applied to MS: automated diagnosis and segmentation, differentiating between subtypes and predicting disease progression. It is apparent that the vast majority of techniques used at the time of publication utilized some variation on U-Net, a type of CNN, as a basis for initial lesion segmentation. It is also clear that unsupervised learning techniques are being explored to limit human bias, a problem that has hindered studies in establishing a ground truth for data. Greater data accessibility would assist in augmenting both the volume and rate of quality research and would allow for increased heterogeneity in datasets for both MS subtypes and imaging quality. The likelihood is that a reliable and generalizable computational framework for MS assessment will increase with greater public access to larger quantities of medical data. Achieving this objective would allow for MS subtypes to be differentiated more easily from one another and for the prediction of disease progression to be estimated with a higher degree of confidence. Such a breakthrough would mean that individuals with the most aggressive forms of MS could be prioritized for earlier and more intensive and focused treatment.

## Figures and Tables

**Figure 9.**
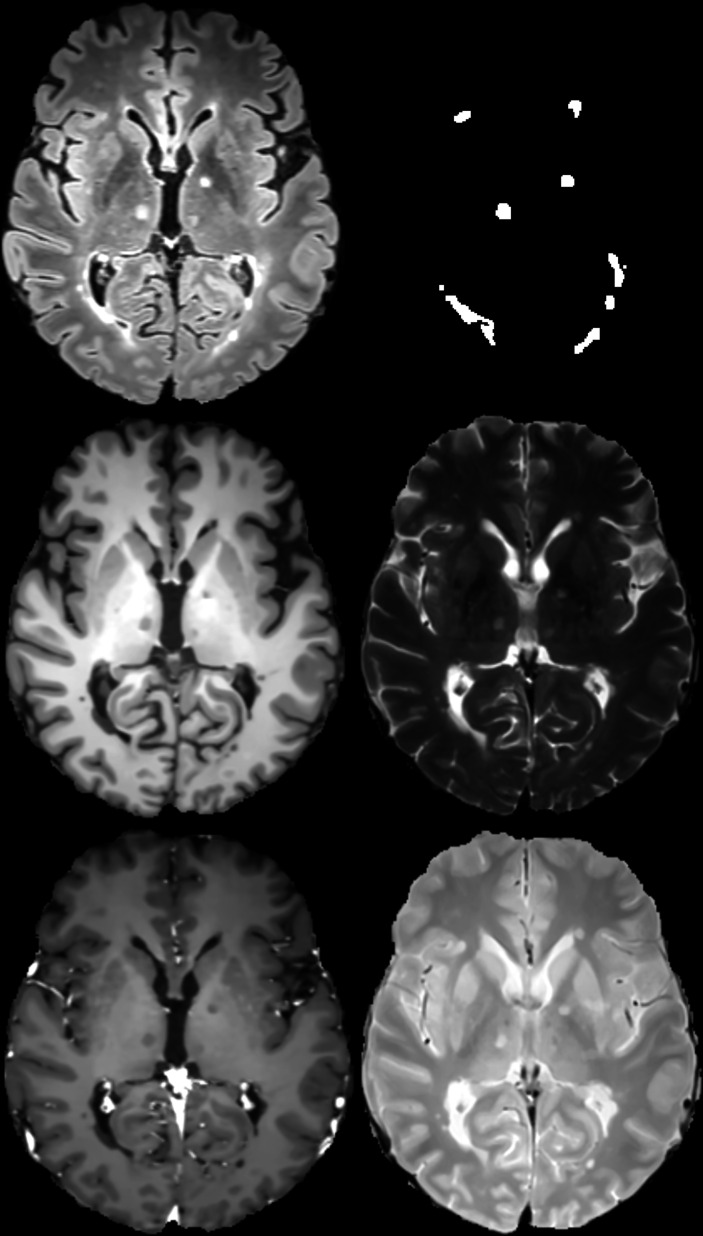
Example MRI scans in the horizontal plane taken from the MICCAI 2016 MS dataset of the same patient using different imaging protocols [204]. From left to right: top row, 1. FLAIR and 2. Consensus position of lesions based upon the manual delineation of seven radiologists; middle row, 3. T1 and 4. T2; bottom row, 5. Gadolinium enhanced and 6. Proton density.

## Data Availability

This article has no additional data.

## Appendices

The appendices outline the few publicly available datasets of MS brain MRI scans paired with ground truth segmentation, as well as published segmentation results for various studies that have participated in the challenge. Appendix A provides context to how lesion segmentation is assessed, both generally and in each of the datasets published. A variety of metrics used to describe and explain lesion segmentation results are provided to assist the reader in understanding the tabulated data in appendix B.

## Appendix A. Evaluation of lesion segmentation

Predictably, the most obvious validation method in determining whether an algorithm accurately counts the number of lesions present on an MRI image is by actually counting. This is known as total lesion load (TLL). This is complemented by the volume difference (VD) metric, which yields the fractional difference between ground truth lesion volume ($LV_{gt}$) and segmentation lesion volume ($LV_{seg}$):

(A1)
$$VD=\frac{\left|LV_{gt}-LV_{seg}\right|}{LV_{gt}}.$$

TLL and VD are both limited performance measures because they do not indicate whether the location of lesions is accurate, nor do they yield any information about lesion size or shape. The most frequently occurring set of metrics used in the literature to address these limiting factors are those relating to the overlap between ground truth data (the point of reference data considered to be real) and the output of the method. They are based on four variables, true positive (TP)—volume of correctly predicted lesion presence; true negative (TN)—volume of correctly predicted lesion absence; false positive (FP)—volume of incorrectly predicted lesion presence; and false negative (FN)—volume of incorrectly predicted lesion absence. The metrics, all of which yield values between zero and one, include *Dice similarity* or *similarity coefficient (DSC*) [269]:

(A2)
$$DSC=\frac{2\times TP}{FP+FN+2\times TP},$$

which is perhaps the standard for assessing segmentation performance and is essentially a measure of similarity between ground truth image and the output produced by a method across all images. For context, a score of 0.7 is considered to be an excellent result in automatic lesion segmentation [270,271] and 0.8 is considered to be near perfect [219]. *Sensitivity*, also known as TPR, is given by

(A3)
$$TPR=\frac{TP}{TP+FN}$$

and describes the fraction of correctly detected lesions. *Specificity* is denoted by

(A4)
$$TNR=\frac{TN}{FP+TN}$$

and indicates the fraction of healthy tissue correctly detected. The positive predictive value (PPV) is described below by

(A5)
$$PPV=\frac{TP}{TP+FP}$$

and reports the percentage of correctly identified lesions over all lesions detected. *Accuracy (Acc*) is given by

(A6)
$$Acc=\frac{TP+TN}{TP+FP+FN+TN}$$

and denotes the fraction of correctly identified tissue. False-positive rate can be calculated through using

(A7)
$$FPR=\frac{FP}{FP+TN}$$

and informs on the percentage of incorrectly detected lesion volume present in all healthy tissue. In addition, in published MS lesion segmentation challenges [131,220,272,273], overall scores achieved by participating algorithms are often calculated based on a weighted amalgamation of all the metrics taken, yielding a comprehensive assessment of a method. Overall scores are the basis for the rankings seen in the tables in subsequent sections, score formulae vary from challenge to challenge. However, metrics can be misleading in cases in which many more lesions are detected than what are present in the ground truth due to small lesion size relative to the quantity of surrounding tissue. This can result in high scores in situations in which algorithms have performed badly [217]. Even when the same metric is employed to assess techniques, it is fundamentally necessary to keep other variables, including image properties and the corresponding masks/labels, the same; otherwise, it can become problematic when comparing the results recorded across papers if the data used are not identical [219,274]. Dice scores for lesion detection have varied between 0.53 and 0.86 [132–151,201,208–211,219,275,276], but different datasets are used within these papers, some of which are publicly available, while others are not. Certain studies focus on detection of all lesions, others solely on white matter lesions and others on cortical lesions. It should be further stressed that the quality of images and masks is crucial, which may explain the large variation in results across segmentation studies using different data repositories and, therefore, the limitations when applied to certain datasets.

In the 2015 ISBI competition [272], DSCs exceeding 0.7 even 7 years after publication have not been achieved across all published entrants [137,139,140,152,153,260,277–280]. Nevertheless, other works have been published, which have yielded significantly better results: 0.86 [211] and 0.78 [150] in which private datasets with restricted access were used. It should be noted that in the best performance, lesions ≤0.2 ml in volume were excluded from the study and that an automatic segmentation prior (MRIAP) was utilized in generating the ground truth before manual experts adjusted them [211]. Ideally, no lesions should be ignored, and all segmentation for the ground truth should be manually performed for validation purposes. It is possible that methods used have improved; it is also, however, plausible that the improvement in scores are a consequence of either larger datasets, better image quality, more homogeneous datasets (i.e. all data produced from the same scanner type) or more precise image segmentation for image labels/masks by radiologists. This is supported by information documented in [153], in which improved results using the same methods were demonstrated with a second dataset for the method described along with a number of others, 0.697 [280], 0.723 [260] and 0.755 [153]. It is therefore imperative that when assessing methods to compare results produced on identical data to ensure results are as a consequence of the properties of the method proposed as opposed to those of the dataset (such as manual segmentation of ground truth or image resolution). This point is most starkly illustrated when examining the works found that ostensibly perform well using the 2016 dataset compared to previous studies published using the same data, achieving Dice scores of 0.824 [132], 0.866 [281] and 0.76 [282]. In all three cases, only the 15 patients from the training set are used in the study for both training and testing. The two-dimensional images comprising the 15 three-dimensional scans are redistributed so that 80% of them are used for training and 20% are used for testing. This breaks the rubric of the challenge, where the challenge states that 15 patients should be used for training and a separate dataset provided comprising 38 patients should be used for testing. It should also be noted that the exclusion of the testing set provided means that data from one of the four centres collected in this study are entirely excluded. As a consequence, this skews the results to make the method used appear better than it actually is and means that the methods are not tested as extensively as they might have been had data from all the scanners collected been used.

## Appendix B. Publicly available magnetic resonance imaging datasets

A variety of MRI sequences exist, each with its own benefits in visualizing tissue types. The most commonly used MRI sequences are T1-weighted, T2-weighted and FLAIR. The two variables that distinguish these from each other are repetition time (TR) and time echo (TE). The former describes the time between externally emitted radio frequency (RF) pulses and the latter is the time between the emission of an RF signal and the reception of an echo. Signal times are shortest in T1 MRI and longest in FLAIR. Differences in appearance between tissues are entirely dependent on water content that dictates the resonant frequency of the tissue. The closer to its resonant frequency a tissue is, the brighter it appears. Based on the research, five publicly available datasets associated with real patients were found. While some early published synthetic data do exist, e.g. BrainWeb [283–286], they will only be briefly mentioned here since their usefulness has been arguably superseded by the resources detailed below.

### B.1. 2008 MICCAI multiple sclerosis lesion segmentation challenge

The MICCAI anonymized dataset comprises 45 3D MRI scans collected by both the University of North Carolina (UNC), Chapel Hill and Boston Children’s Hospital (BCH). 3T Siemens scanners were used in both instances [220]. Only two experts manually segmented the images from UNC, and just one expert segmented those provided by BCH. Multiple MRI modalities were used for each patient: T1, T2, FLAIR, DTI-derived fractional anisotropy and mean diffusivity images. Resolution was $0.5\;\times \;0.5\;\times \;0.5\;mm^{3}$ [220]. The training portion of the dataset was composed of 20 patients, all of which underwent a 3D scan, 10 from each respective scanning centre, whereas 25 cases make up the testing set, 15 from CHB and 10 from UNC. The dataset can be accessed through the *NeuroImaging Tools and Resources Collaboratory* website: https://www.nitrc.org/projects/msseg (last accessed: 19 February 2023).

An up-to-date leaderboard can be found at the following address: http://www.ia.unc.edu/MSseg/results_table.php (last accessed: 19 February 2023). Only results associated with a publication were included in table 1. Best results of each participant were recorded (two of the three best results were produced by [140]).

**Table 1.** Results using 2008 MICCAI MS lesion segmentation dataset. VD = volume difference; TPR = true positive rate; FPR = false positive rate.

	UNC rater			CHB rater			overall	
method	VD	TPR	FPR	VD	TPR	FPR	score	rank
[140]	62.5	55.5	46.8	40.8	68.7	46.0	87.12	1
[287]	46.9	43.9	32.3	113.4	53.5	24.2	86.93	2
[141]	46.3	47.0	43.5	51.3	52.7	42.0	86.11	3
[154]	37.8	42.0	44.1	53.4	51.8	45.1	84.46	4
[147]	64.7	71.8	69.1	118.6	84.0	69.7	84.34	5
[288]	58.1	59.0	64.7	96.8	71.3	62.8	84.16	6
[289]	33.1	63.8	69.7	59.3	68.0	68.6	84.25	7
[152]	63.5	47.1	52.7	52.0	56.0	49.8	84.07	8
[290]	56.9	37.7	34.6	113.7	42.9	30.5	83.92	9
[155]	88.6	55.5	62.6	45.1	68.1	63.2	83.25	12
[156]	65.2	44.9	43.2	158.9	55.4	40.5	82.34	14
[157]	49.0	39.7	60.8	73.0	47.9	52.7	82.16	15
[158]	45.4	51.2	76.7	52.4	59.0	71.5	82.07	16
[159]	57.9	49.1	76.3	86.4	58.2	70.6	80.00	22
[201]	63.7	49.0	74.9	85.2	55.8	70.5	79.90	24
[144]	96.0	48.4	83.6	46.4	60.1	81.2	78.19	30
[291]	183.7	20.0	18.0	387.5	24.6	18.2	77.96	31

### B.2. 2012 Institute of Neurology and Genetics Nicosia, Cyprus, multiple sclerosis dataset

The dataset is longitudinal with each of 38 patients being scanned twice with an interval of between 6 and 12 months. At the time of the initial scan, each of the patients had to be diagnosed with CIS. The gender distribution was approximately even, with 17 men and 21 women. A T2 scanning sequence was used with the repetition time (TR) = 4408 ms and echo time (TE) = 100 ms, a slice thickness of 5 mm, a field of view of 230 mm and pixel resolution of 2.226 mm^−1^. In addition, 10 healthy candidates were included as controls. All lesions thought to be clinically significant were delineated by a neurologist and validated by a radiologist [292–295]. The dataset can be located and freely downloaded from the *Department of Computer Science, University of Cyprus* (Nicosia) website: http://ehealthlab.cs.ucy.ac.cy/index.php/32-software/218-datasets (last accessed: 19 February 2023).

### B.3. 2015 Longitudinal multiple sclerosis lesion segmentation challenge

The dataset was published in association with the ISBI [271,296]. The data were collected from University College Hospital, London. The data repository was separated into training and testing data of 5 and 14 subjects, respectively. The dataset was longitudinal. Four of the five participants in the training set were scanned at four points in time, and the remaining patient was scanned at five [272]. The test data were subdivided into set A, comprising ten subjects, and set B, comprising four subjects. In set A, eight of the subjects were scanned four times and two were scanned five times. In set B, three patients were scanned four times, and one patient was scanned five times. Four different scanning modalities were used for each patient: T1-weighted (TR = 10.3 ms, TE = 6 ms, res = 0.82 × 0.82 × 1.17 mm${,}^{3}$), double spin-echo PD-weighted (TR = 4177 ms, TE = 12.31 ms, res = 0.82 × 0.82 × 2.2 mm${,}^{3}$), T2-weighted (TR = 4177 ms, TE = 80 ms, res = 0.82 × 0.82 × 2.2 mm${,}^{3}$) and T2-weighted FLAIR (inversion time (TI) = 835 ms, TE = 68 ms, res = 0.82 × 0.82 × 2.2 mm${,}^{3}$) all using a 3T Philips system [272,296]. Two raters were used in the manual delineation of all images, one of whom had 4 years of experience and the other had 10 [272]. The ratio of subjects is skewed both in favour of women (15:4) and relapsing–remitting patients (16:3); notably, no male primary progressive patients are present in the dataset. The dataset can be downloaded from the *smart-stats tools website on the longitudinal MS lesion segmentation challenge* webpage: https://smart-stats-tools.org/lesion-challenge-2015.

Table 2 illustrates a selection of published results found using the dataset. The leaderboard can be found at the following address: https://smart-stats-tools.org/lesion-challenge. Only results associated with a publication were included in table 2.

**Table 2.** Results using 2015 ISBI longitudinal MS lesion segmentation dataset. DSC = Dice score; PPV = positive predictive value; TPR = true positive rate; LFPR = lesion false positive rate; LTPR = lesion true positive rate; VD = volume difference.

method	DSC	PPV	TPR	LFPR	LTPR	VD	score	rank
[297]^a^	0.669	0.886	0.575	0.125	0.538	0.396	93.358	1
[153]	0.639	0.914	0.530	0.122	0.533	0.436	93.317	2
[297]	0.643	0.908	0.533	0.124	0.520	0.428	93.210	3
[151]	0.655	0.886	0.555	0.140	0.544	0.399	93.238	4
[243]	0.681	0.855	0.603	0.157	0.538	0.366	93.030	5
[280]	0.687	0.849	0.615	0.168	0.554	0.359	93.087	6
[298]	0.643	0.887	0.546	0.132	0.480	0.421	92.730	7
[299]	0.643	0.879	0.534	0.105	0.441	0.399	92.637	8
[300]	0.662	0.838	0.590	0.151	0.491	0.372	92.554	9
[277]	0.584	0.921	0.456	0.414	0.087	0.497	92.486	10
[137]	0.611	0.899	0.490	0.410	0.139	0.454	92.118	11
[160]	0.630	0.845	0.537	0.487	0.201	0.405	92.076	12
[139]^a^	0.639	0.813	0.567	0.120	0.366	0.356	91.633	13
[138]	0.631	0.787	0.555	0.367	0.153	0.338	91.331	14
[301]	0.633	0.830	0.548	0.242	0.347	0.412	90.591	15
[302]	0.605	0.775	0.535	0.266	0.367	0.365	90.283	16
[139]	0.627	0.789	0.555	0.498	0.568	0.352	90.070	17
[303]	0.568	0.611	0.570	0.474	0.353	0.343	87.72	18
[304]	0.501	0.549	0.523	0.577	0.429	0.571	86.92	19

^a^ Submitted results that improved on those associated with publications.

### B.4. 2016 MICCAI multiple sclerosis lesion segmentation challenge

This is the second dataset published by MICCAI, comprising 53 individuals with MS [131,305]. The data were collected from various hospitals across France using different MRI scanners produced by different manufacturers: centre 01, University Hospital of Rennes (Siemens Verio 3T); centre 03 University Hospital of Bordeaux (General Electrics Discovery 3T); centre 07, University Hospital of Lyon (Siemens Aura 1.5T) and centre 08, University Hospital of Lyon (Philips Ingenia 1.5T). Centres 01, 07 and 08 contributed five patients each to the training data—giving a total of 15 patients—and 10 patients each to the testing data; centre 03 also provided an additional eight patients, giving a total of 38 patients in the testing dataset [305]. Sample scans of the various imaging protocols can be seen in figure 9. Although the dataset comprises 3D scans, the images are shown in the horizontal plane as opposed to sagittal or coronal planes, since lesions appear most conspicuous from this angle and are thus most informative for the purpose of this review.

The gender ratio was 38:15 in favour of women across all centres. This dataset includes a wide range of MRI types. FLAIR, T1, T2, PD and Gd-enhanced MRI scans are included. TE, TR and resolutions vary from both centre to centre and scan type to scan type; see the reference paper for further details [305]. Seven experts (junior radiologists) delineated the images based on both the T2 and FLAIR images; consensus segmentation masks were then generated using iterative software, using the combined delineation performance of each of the experts [305]. Lesions smaller than 3 mm${,}^{3}$ were removed as they were considered to be an unreliable basis for true lesions [100]. The dataset is downloadable after setting up an account on the *Shanoir platform* using the following webpage: https://shanoir.irisa.fr/shanoir-ng/challenge-request.

No leaderboard of results is publicly accessible; therefore, there may be discrepancies across the different studies so that direct comparison may or may not be appropriate. Not all participants of the challenge published their results explicitly, although they were plotted on a graph by the event organizers, giving an indication of the most effective entrees [131]. Results found are displayed in table 3.

**Table 3.** Results using 2016 MICCAI MS lesion segmentation dataset. DSC = Dice; TPR = true positive rate; PPV = positive predictive value.

method	DSC	TPR	PPV
[306]	0.566	0.456	0.287
[307]	0.571	0.358	0.526
[308]	0.60	0.53	0.80
[309]	0.502	X	X
[310]	0.651	0.689	X
[311]^a^	0.63	0.57	X
[311]^b^	0.60	0.69	X
[281]^c^	0.866	0.856	X
[132]^c^	0.824	0.762	X
[280]^c^	0.76	0.65	X

^a^ LeMan-PV method.

^b^ PV-CNN method.

^c^ Studies that use dataset but do not adhere to the set.

### B.5. 2021 MICCAI multiple sclerosis lesion segmentation challenge

The 2021 MICCAI dataset was the largest to ever be made publicly available. It comprises 100 MS patients, 40 for training and 60 for testing and the FLAIR MRI sequence was used in its creation. The dataset is longitudinal, with two scans associated with each patient, and the follow-up scan collected between 1 and 3 years after the initial one [273]. A total of 15 different MRI scanners were used in the data collection process: three General Electric, six Philips and six Siemens. Lesion segmentation of ground truth for each MRI scan was performed by four neuroradiologists, then a consensus was formed under the guidance and supervision of an expert neuroradiologist. Like the previous dataset, it is downloadable after setting up an account on the *Shanoir platform* using the same webpage: https://shanoir.irisa.fr/shanoir-ng/challenge-request.

No leaderboard of results is publicly accessible; therefore, there may be discrepancies across the different studies so that direct comparison may or may not be appropriate. Furthermore, at the time of writing no analysis has been published with respect to the results of the challenge. Published results found associated with the dataset can be seen in table 4.

**Table 4.** Results using 2021 MICCAI MS lesion segmentation dataset. DSC = Dice; TPR = true positive rate.

method	DSC	TPR	accuracy
[312]	0.557	X	X
[313]	0.75	X	X
[314]	0.728	0.666	X
[315]	0.437	X	0.65
[316]	0.62	0.58	X
[317]	0.54	0.58	X
[318]	0.36	0.75	X
[319]	0.743	X	X
[320]	0.564	X	X
[321]	0.508	X	X
[322]	0.451	0.718	X
